# Global trends in burden of type 2 diabetes attributable to physical inactivity across 204 countries and territories, 1990-2019

**DOI:** 10.3389/fendo.2024.1343002

**Published:** 2024-02-26

**Authors:** Xinyue Yang, Jiayi Sun, Wenjuan Zhang

**Affiliations:** Department of Cardiovascular Medicine, Tianjin Medical University General Hospital, Tianjin, China

**Keywords:** global burden, type 2 diabetes, physical inactivity, mortality, disability-adjusted life years, sociodemographic index

## Abstract

**Background:**

To promote a comprehensive understanding of global trends and burden of type 2 diabetes attributable to physical inactivity.

**Methods:**

We utilized data regarding mortality, disability-adjusted life years (DALYs), as well as age-standardized mortality rates (ASMR) and DALYs rates (ASDR) derived from the global burden of disease study 2019 to evaluate the impact of physical inactivity on the prevalence of type 2 diabetes in 204 countries and territories over the period from 1990 to 2019. This method facilitated the analysis of the diabetes burden across different ages, genders, and regions. To determine the long-term progression of type 2 diabetes prevalence, we computed the estimated annual percentage change (EAPC) in burden rates.

**Results:**

Globally, the number of deaths and DALYs from type 2 diabetes due to physical inactivity more than doubled between 1990 and 2019. Concurrently, there was an increase in the ASMR and ASDR, with EAPC of 0.26 (95% CI: 0.13-0.39) and 0.84 (95% CI: 0.78-0.89), respectively. As of 2019, the global ASMR and ASDR for physical inactivity stood at 1.6 (95% UI: 0.8-2.7) per 100 000 and 55.9 (95% UI: 27.2-97.6) per 100 000, respectively. Notable disparities were observed in the type 2 diabetes burden associated with physical inactivity worldwide, with higher sociodemographic index (SDI) countries experiencing lower ASDR and ASMR compared to lower SDI countries. Initially, females exhibited higher ASMR and ASDR than males, but this gender disparity in ASMR and ASDR has lessened in recent years. The mortality and DALYs rates associated with physical inactivity exhibit an inverted V-shaped pattern across various age groups, predominantly affecting the elderly population.

**Conclusion:**

Between 1990 and 2019, there was a marked rise in the worldwide burden of type 2 diabetes associated with physical inactivity, underscoring the role of physical inactivity as a key changeable risk factor in the global landscape of this disease. This necessitates additional research to explore the variables contributing to the varying levels of disease burden across different countries and between sexes. Furthermore, it calls for the formulation of public health policies aimed at guiding prevention tactics, promoting early detection, and enhancing the management of type 2 diabetes.

## Introduction

Over the past few decades, there has been a significant increase in diabetes cases, closely linked to changes in lifestyle habits (1). The International Diabetes Federation reports that in 2021, about 536.6 million adults aged 20-79 were living with diabetes, and this number is expected to surge to 783.2 million by 2045 (2). Diabetes poses a substantial challenge to public health, profoundly affecting individuals, families, and communities worldwide (3). Type 2 diabetes, which accounts for over 90% of all diabetes cases globally, is a metabolic condition marked by insufficient insulin production and resistance, leading to high blood sugar levels. The 2019 global burden of disease (GBD) study provides recent data, estimating that the worldwide prevalence, mortalities, and disability-adjusted life years (DALYs) due to type 2 diabetes were approximately 437.9 million, 1.5 million, and 66.3 million, respectively (4).

Four major non-communicable diseases (cardiovascular diseases, diabetes, cancer and chronic respiratory disease) cause just over 70% of all preventable deaths occurring worldwide, around 41 million people (5). Tobacco, physical inactivity, alcohol abuse, and unhealthy diets increase the risk of dying from non-communicable diseases (6). As of 2016, it was estimated that about 27.5% of the global population exhibited insufficient physical activity, with a 95% uncertainty interval (UI) of 25.0-32.2 (7). Recognized as a worldwide health crisis, physical inactivity significantly contributes to the overall burden of disease. In 2008, lack of physical activity was estimated to be responsible for approximately 9% of premature mortalities worldwide, equating to over 5.3 million out of the total 57 million deaths (8). Moreover, the economic impact of physical inactivity is substantial. In 2013, it led to a global loss of productivity amounting to $13.7 billion (9).

Despite physical inactivity being recognized as a key modifiable risk factor for type 2 diabetes (10, 11), global epidemiological investigations of this association remain limited, often concentrating on smaller developed country samples failing to reflect worldwide trends comprehensively. However, physical activity levels differ globally, potentially impacting regional type 2 diabetes burdens. Therefore, this study utilizes global epidemiological data to evaluate changes in type 2 diabetes burden attributable to physical inactivity across 204 countries/territories from 1990-2019, informing targeted diabetes prevention and control strategies globally.

## Methods

### Data source

This research utilized data from the GBD 2019. The GBD estimation methodology involves identifying multiple relevant data sources for each disease or injury, including censuses, surveys, civil registries, disease registries, health services, environmental monitors, imaging, notifications, and others. These are identified via systematic reviews of published studies, government/organizational websites, reports, and GBD collaborator datasets. Each newly identified and obtained source receives a unique identifier from the librarian team and inclusion into the Global Health Data Exchange (GHDx: https://ghdx.healthdata.org/). The GHDx publicly provides the metadata and available data for all sources used in GBD estimates, allowing to identify the specific sources used to estimate any disease/injury outcome (12, 13).

Our approach adhered to the methodological principles and analytic techniques specified in GBD 2019, which are elaborated in other publications (14). This initiative offered epidemiological insights and quantifications for 369 diseases and injuries in 204 countries and territories spanning from 1990 to 2019 (13). Utilizing comprehensive datasets across different ages, time periods, regions, and health categories, the GBD network employed uniform Bayesian methods to produce disease-specific estimates. This integration of diverse data sources with modeling techniques enabled the estimation of global disease burden across nations and regions despite gaps in population health information (12).

In the GBD 2019, risk factors are stratified into four tiers within a causality framework, ranging from the most general (Level 1, such as NCDs) to the most specific (Level 4, like type 2 diabetes), to gauge their impact on disease burden (15). For diagnosing type 2 diabetes, the primary benchmark is fasting plasma glucose levels above 126 mg/dl or ongoing treatment with medication or insulin for T2DM. Yet, in the GBD 2019, additional indicators such as oral glucose tolerance, and postprandial glucose tests were also recognized, albeit varying from the main diagnostic criteria (4). Consequently, these alternate diagnostic approaches, serving as data inputs, underwent adjustment before the start of the modeling phase. Physical inactivity, categorized as Level 2, was defined as less than 3,000-4,500 metabolic equivalent (MET) minutes per week, averaged across occupational, household, transport, and leisure activities. The MET minutes per week was calculated as follows: minutes of activity/day × days per week × MET level (16). Data on physical inactivity were compiled from 376 sources worldwide (17).

We sourced data on the impact of physical inactivity on type 2 diabetes globally from 1990 to 2019, by country, region, and gender, from the GHDx. These data encompassed 204 countries and territories, divided into five groups based on the sociodemographic index (SDI): high, high-middle, middle, low-middle, and low. SDI, a measure of regional development, is calculated from the total fertility rate among females under 25, education attainment for individuals aged 15 and older, and the lagged per capita gross domestic product, with values ranging from 0 (least developed) to 1 (most developed) (12). Furthermore, these countries were grouped into 21 geographical regions (**Table 1**). For detailed analysis, age was segmented into 15 categories, including fourteen 5-year intervals from 25-94 years and one category for ages ≥95 years.

**Table 1:** The global type 2 diabetes burden attributable to physical inactivity in 1990 and 2019 and the temporal trends from 1990 to 2019.

characteristic	1990				2019				EAPC (1990-2019)	
	Death cases, n × 10^3^ (95% UI)	ASMR per 10^5^, n (95% UI)	DALYs, n × 10^3^ (95% UI)	ASDR per 10^5^, n (95% UI)	Death cases, n × 10^3^ (95% UI)	ASMR per 10^5^, n (95% UI)	DALYs, n × 10^3^ (95% UI)	ASDR per 10^5^, n (95% UI)	ASMR, n (95% CI)	ASDR, n (95% CI)
**Global**	49.8 (24.5-84.6)	1.5 (0.8-2.5)	1719.8 (782-3071.2)	45 (21.3-79.5)	125.2 (62.1-208.3)	1.6 (0.8-2.7)	4549.2 (2188.5-7969.5)	55.9 (27.2-97.6)	0.26 (0.13-0.39)	0.84 (0.78-0.89)
Sex										
Male	18.9 (8.5-33.8)	1.4 (0.6-2.4)	691.5 (288.5-1292.4)	40.5 (17.9-73.9)	54 (25.7-91.5)	1.6 (0.8-2.7)	2038.8 (898.4-3704.7)	54.3 (25-97.6)	0.51 (0.37-0.65)	0.99 (0.93-1.05)
Female	30.9 (16-50.9)	1.6 (0.8-2.6)	1028.3 (500.4-1788.9)	49.1 (24.1-84.6)	71.2 (37-115.6)	1.6 (0.8-2.6)	2510.4 (1251.7-4297.5)	57.5 (28.6-98.4)	0.09 (-0.04-0.23)	0.73 (0.66-0.79)
Socio-demographic index										
High SDI	12.7 (6.1-21.3)	1.2 (0.6-2)	420.3 (187.2-765.9)	40.5 (17.8-74.8)	19.6 (9.6-32.2)	0.9 (0.4-1.5)	879 (410.8-1574.4)	49.7 (23-91.8)	-1.48 (-1.81–1.15)	0.63 (0.54-0.73)
High-middle SDI	12.6 (6.5-20.3)	1.4 (0.7-2.2)	445.7 (213.5-759)	43 (21.1-72.5)	25.6 (13.4-40.5)	1.3 (0.7-2)	963.1 (481.4-1662.4)	47.6 (23.8-81.9)	-0.2 (-0.33–0.07)	0.39 (0.29-0.49)
Middle SDI	13.1 (6.1-22.9)	1.7 (0.8-2.8)	466.9 (208.9-839.4)	48.1 (22.4-84.4)	44.2 (21.6-74.5)	2.1 (1-3.5)	1545.7 (707.7-2717.5)	63.8 (30-110.3)	0.89 (0.82-0.96)	1.04 (0.99-1.09)
Low-middle SDI	8.2 (4.1-14.2)	1.9 (1-3.2)	280.2 (131.6-505)	51.3 (25.5-90.2)	27.4 (14-45.5)	2.5 (1.3-4.1)	872.1 (429.4-1524.2)	68.1 (34.2-115.4)	1.07 (0.91-1.24)	1.19 (1.09-1.29)
Low SDI	3.1 (1.4-5.9)	1.8 (0.9-3.2)	104.5 (44.7-200.8)	47.9 (21.8-88.1)	8.2 (3.9-14.3)	2.1 (1-3.6)	283.5 (125.3-520.1)	58.5 (27.3-105.9)	0.73 (0.56-0.89)	0.96 (0.84-1.09)
Region										
Andean Latin America	0.2 (0.1-0.3)	1 (0.4-1.8)	5.5 (2-11.2)	27.9 (10.2-55.8)	0.9 (0.4-1.6)	1.7 (0.7-3.1)	28.4 (11.3-54.8)	51.5 (20.7-98.6)	2.1 (1.93-2.26)	2.18 (2.1-2.26)
Australasia	0.3 (0.1-0.5)	1.3 (0.7-2.1)	8.5 (3.9-14.8)	36.7 (16.5-64.1)	0.7 (0.4-1.1)	1.3 (0.7-2)	25.6 (12.6-43.2)	52.8 (25.3-91.2)	-0.49 (-0.84–0.13)	1.08 (0.92-1.25)
Caribbean	1.3 (0.7-2)	5.3 (2.9-8.3)	40.8 (19.7-69.1)	157.9 (77.2-265.7)	2.6 (1.4-4)	5 (2.7-7.8)	95.2 (48.1-160.7)	184.1 (93.5-310.6)	-0.3 (-0.37–0.22)	0.47 (0.41-0.53)
Central Asia	0.2 (0.1-0.3)	0.4 (0.2-0.7)	7.9 (3.3-15.7)	17.4 (7.6-33.6)	0.8 (0.3-1.4)	1.3 (0.6-2.3)	31.4 (13.2-62.2)	44.9 (19.8-86.4)	3.78 (3.47-4.08)	3.18 (3.01-3.36)
Central Europe	1 (0.5-1.8)	0.7 (0.4-1.3)	45.1 (20.1-84.3)	31 (14.1-58.5)	2 (1-3.5)	0.9 (0.4-1.5)	90.8 (42.4-166.9)	42.9 (19.6-79.5)	1.03 (0.86-1.19)	1.3 (1.13-1.47)
Central Latin America	2.1 (0.9-4)	2.8 (1.2-5.3)	74.8 (28.3-147.1)	88.6 (34.4-171.1)	6.7 (2.8-12.5)	3 (1.2-5.5)	224.2 (85.6-435.2)	95.1 (36.6-184)	-0.13 (-0.34-0.08)	0.21 (0-0.43)
Central Sub-Saharan Africa	0.5 (0.2-1)	3 (1.3-5.6)	16.6 (6.4-34.5)	77.2 (31.7-152.7)	1.2 (0.5-2.2)	3.1 (1.4-5.5)	44.9 (17.4-90.2)	86.5 (35.7-163.8)	-0.05 (-0.1-0)	0.35 (0.28-0.42)
East Asia	4.6 (2.1-8.4)	0.7 (0.3-1.2)	192.1 (83.7-357.3)	24.3 (11-43.1)	13.2 (6.1-23.2)	0.8 (0.4-1.3)	487.2 (208.5-922.4)	24.6 (10.9-45.8)	-0.03 (-0.24-0.18)	-0.37 (-0.54–0.21)
Eastern Europe	0.5 (0.2-0.9)	0.2 (0.1-0.4)	28.6 (12.6-54.2)	10.6 (4.7-20)	1.4 (0.6-2.5)	0.4 (0.2-0.7)	56.1 (25.8-102.9)	15.8 (7.3-29.5)	2.39 (1.72-3.06)	1.69 (1.49-1.9)
Eastern Sub-Saharan Africa	0.5 (0.2-1.2)	1 (0.4-2)	14.6 (5.5-32.3)	21.6 (8.3-47.3)	1.1 (0.4-2.3)	1 (0.4-2)	30.6 (11.6-68.1)	21.4 (8.2-45)	-0.1 (-0.14–0.06)	-0.08 (-0.11–0.05)
High-income Asia Pacific	1.4 (0.6-2.5)	0.8 (0.4-1.4)	64.2 (25.2-123.5)	32.1 (12.7-61.4)	2.5 (1.2-4.2)	0.4 (0.2-0.8)	132.1 (56.1-245.3)	31.8 (12-63)	-2.14 (-2.31–1.98)	-0.36 (-0.48–0.24)
High-income North America	4.6 (2.1-7.9)	1.3 (0.6-2.2)	160.6 (69-298.6)	46.3 (19.5-86.9)	6.3 (2.8-11.3)	0.9 (0.4-1.7)	293.4 (122.9-566.7)	49 (20.4-96)	-1.67 (-2.29–1.05)	0.5 (0.28-0.72)
North Africa and Middle East	4.9 (2.7-7.9)	3.6 (2-5.6)	179.4 (89.6-298.6)	106.4 (56.1-173.2)	14.4 (8.1-22.1)	3.9 (2.2-5.9)	685 (356.4-1111.5)	154 (82.7-245.6)	0.51 (0.36-0.65)	1.53 (1.4-1.66)
Oceania	0.2 (0.1-0.3)	7.4 (3.5-13.3)	5.3 (2.2-10.5)	182.2 (80.1-342.3)	0.6 (0.3-1.1)	11 (5.3-19.3)	20 (8.3-38.5)	281.4 (125.3-525.6)	1.14 (0.86-1.42)	1.32 (1.03-1.61)
South Asia	7.2 (3.5-12.8)	2 (1-3.4)	251.8 (113.6-461.3)	51.5 (24.8-91.5)	26.2 (13.1-43.7)	2.5 (1.3-4.1)	783.8 (374.1-1426.2)	62.2 (30.7-109.7)	0.95 (0.6-1.3)	1.14 (0.9-1.38)
Southeast Asia	3.8 (1.6-7.6)	1.9 (0.8-3.6)	112.7 (43.8-228.1)	47.4 (19.6-94.3)	12.9 (5.5-24.2)	2.5 (1.1-4.6)	403.3 (160.6-772.6)	69 (28.8-130.6)	0.96 (0.9-1.03)	1.25 (1.17-1.33)
Southern Latin America	0.2 (0.1-0.4)	0.5 (0.2-1)	6.1 (2.3-13.2)	13.4 (5-28.7)	0.6 (0.2-1.1)	0.7 (0.3-1.2)	20.1 (8.3-39.5)	24.1 (10-47.7)	1.26 (1-1.52)	2.31 (2.09-2.54)
Southern Sub-Saharan Africa	1 (0.5-1.6)	4 (1.9-6.6)	28.9 (13.4-50.3)	105.3 (49.5-180.1)	3.1 (1.5-5.2)	6.7 (3.2-11.2)	86.9 (39.3-151.7)	160.8 (73.5-275.9)	2.43 (1.81-3.05)	2 (1.45-2.55)
Tropical Latin America	4.3 (2.5-6.3)	5.4 (3.2-7.9)	171.7 (93.7-263.8)	181 (101.5-273.5)	11.5 (6.9-16.5)	5 (3-7.1)	429.1 (248.4-650.9)	176 (101.6-266)	-0.24 (-0.3–0.17)	0.01 (-0.04-0.06)
Western Europe	9.5 (4.8-15.3)	1.6 (0.8-2.6)	268.5 (127.2-460.6)	46.1 (21.2-79.8)	13 (6.7-20.6)	1.1 (0.6-1.8)	476.4 (227.2-827.1)	54.1 (24.7-98)	-1.52 (-1.72–1.32)	0.22 (-0.02-0.45)
Western Sub-Saharan Africa	1.3 (0.6-2.4)	1.9 (0.8-3.5)	35.8 (15-70)	44.4 (19.3-84.7)	3.6 (1.5-6.5)	2.6 (1.1-4.5)	104.9 (43.2-200.9)	61.3 (26.2-113.4)	1.01 (0.81-1.21)	1.06 (0.89-1.24)

ASMR, age-standardized mortality rate; ASDR, age-standardized disability-adjusted life year rate; EAPC, estimated annual percentage change.

### Statistical analysis

To assess the global, regional, and national burden of type 2 diabetes due to physical inactivity, we utilized metrics including deaths, age-standardized mortality rates (ASMR), DALYs, and age-standardized disability rates (ASDR). The estimated annual percentage change (EAPC) was employed to analyze the trends in age-standardized rates (ASR) from 1990 to 2019 (18). Trends in ASR were categorized as increasing, decreasing, or stable based on whether the EAPC and its 95% CI were greater than 0, less than 0, or included 0, respectively (19). These analyses were performed using the R software (version 4.0.3).

## Results

### Type 2 diabetes deaths and ASMR associated with physical inactivity

**Figure 1:**
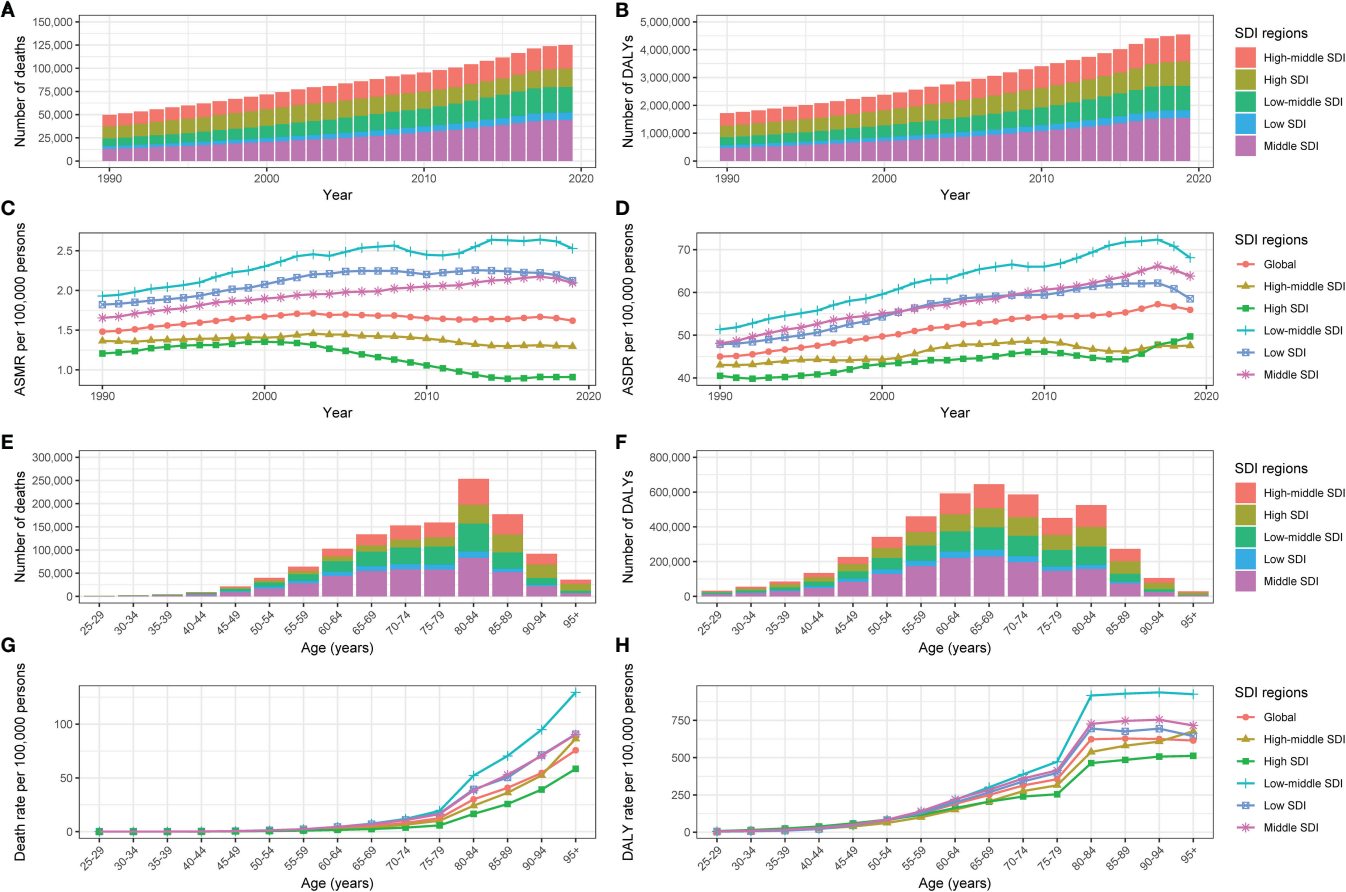
The type 2 diabetes burden attributable to physical inactivity by SDI region. The global **(A)** deaths, **(B)** DALYs, **(C)** ASMR and **(D)** ASDR of type 2 diabetes attributable to physical inactivity for all ages from 1990 to 2019. The global **(E)** deaths, **(F)** DALYs, **(G)** mortality rate and **(H)** DALYs rate of type 2 diabetes attributable to physical inactivity by age in 2019. SDI, Socio-demographic Index; ASMR, age-standardized mortality rate; DALYs, disability-adjusted life years; ASDR, age-standardized DALY rate.

Globally, the number of type 2 diabetes deaths associated with physical inactivity markedly rose from 49.8 thousand (95% UI: 24.5-84.6) in 1990 to 125.2 thousand (95% UI: 62.1-208.3) in 2019 (**Table 1**, **Figure 1A**). Despite this rise in absolute numbers, the global ASMR showed a slight increase from 1.5 (95% UI: 0.8-2.5) to 1.6 (95% UI: 0.8-2.7) per 100 000, with an EAPC of 0.26 (95% CI: 0.13-0.39), indicating a modest rise in the mortality rate over the three decades (**Table 1**, **Figure 1C**).

There was a significant gender difference observed. The number of male fatalities attributed to type 2 diabetes due to physical inactivity increased from 18.9 thousand (95% UI: 8.5-33.8) in 1990 to 54 thousand (95% UI: 25.7-91.5) in 2019, and female deaths rose from 30.9 thousand (95% UI: 16-50.9) to 71.2 thousand (95% UI: 37-115.6). In males, the ASMR modestly increased from 1.4 to 1.6 per 100 000, whereas it remained relatively stable at 1.6 per 100 000 in females. A more pronounced EAPC was observed in males (0.51; 95% CI: 0.37-0.65) compared to females (0.09; 95% CI: -0.04-0.23), as shown in **Table 1**.

**Figure 2:**
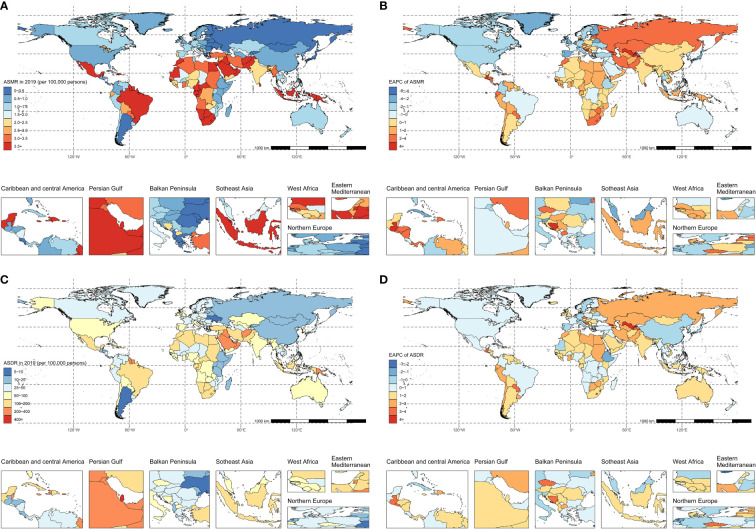
The spatial distribution of type 2 diabetes **(A)** ASMR, **(B)** the EAPC of ASMR, **(C)** ASDR, and **(D)** the EAPC of ASDR attributable to physical inactivity in 2019. ASMR, age-standardized mortality rate; EAPC, estimated annual percentage change; ASDR, age-standardized disability-adjusted life-years rate.

Regarding the SDI, high SDI regions witnessed a decrease in ASMR from 1.2 (95% UI: 0.6-2) per 100 000 in 1990 to 0.9 (95% UI: 0.4-1.5) per 100 000 in 2019, reflecting a significant reduction in mortality with an EAPC of -1.48 (95% CI: -1.81–1.15). The high-middle SDI regions observed a relatively constant ASMR, shifting marginally from 1.4 (95% UI: 0.7-2.2) per 100 000 to 1.3 (95% UI: 0.7-2) per 100 000 over the period 1990-2019, with an EAPC of -0.2 (95% CI: -0.33–0.07). Conversely, the low-middle SDI regions experienced the most notable ASMR increase, from 1.9 (95% UI: 1-3.2) per 100 000 in 1990 to 2.5 (95% UI: 1.3-4.1) per 100 000 in 2019, and an EAPC of 1.07 (95% CI: 0.91-1.24) (**Table 1**, **Figure 1C**). In the GBD regions, South Asia recorded the highest mortality in 2019 with 26.2 thousand deaths (95% UI: 13.1-43.7). The top ASMR in 2019 were in Oceania (11 per 100 000; 95% UI: 5.3-19.3) and Southern Sub-Saharan Africa (6.7 per 100 000; 95% UI: 3.2-11.2) (**Table 1**, **Figure 2A**). ASMR showed declining trends in regions like High-Income Asia Pacific and High-Income North America, while remaining stable in East Asia. However, ASMR were on the rise in Southern Sub-Saharan Africa and Central Asia, with Central Asia experiencing the sharpest increase (EAPC: 3.78; 95% CI: 3.47-4.08) (**Table 1**, **Figure 2B**).

Nationally, India recorded the highest count of type 2 diabetes fatalities attributable to physical inactivity, with numbers reaching 5238.9 (95% UI: 2483.5-9416) in 1990 and escalating to 19070.6 (95% UI: 9589.6-32291.6) by 2019. Qatar had the most elevated ASMR in 1990 at 19.3 (95% UI: 11.1-29) per 100 000, while Fiji reported the highest in 2019 at 29 (95% UI: 14.8-49.6) per 100 000. Conversely, Guatemala exhibited the lowest ASMR in 1990 (0.1 per 100 000; 95% UI: 0.1-0.4), and Ukraine had the lowest in 2019 (0.1 per 100 000; 95% UI: 0.1-0.2). Among 121 countries that experienced an increase in ASMR, Uzbekistan showed the most rapid growth (EAPC: 5.85; 95% CI: 5.26-6.44) (**Figures 2A, B**; **Supplementary Table 1**).

In 2019, the pattern of type 2 diabetes deaths due to physical inactivity in different age groups formed an inverted V-shape, with its peak in the 80-84 age group. The majority of these deaths were among individuals aged 60-89, predominantly in the middle and low-middle SDI regions (**Figure 1E**). Mortality rates in different age groups for patients with type 2 diabetes due to physical inactivity escalated with advancing age, particularly post the age of 80 (**Figure 1G**; **Supplementary Figure 1A**). On a global scale, the EAPC in mortality rates exhibited an inverted U-shaped relationship with age. Negative EAPC values were observed in the 25-29 and 70-74 age groups, while positive values were seen in the 30-64 and 75+ age groups. The sharpest decline in mortality was in the 25-29 age group, and the most significant increase was in the 40-44 age group. However, in low SDI regions, mortality rates rose consistently across all age groups (**Supplementary Figure 1B**).

### Type 2 diabetes DALYs and ASDR associated with physical inactivity

Globally, the number of DALYs for type 2 diabetes associated with physical inactivity more than doubled from approximately 1.7 million (95% UI: 0.8-3.1) in 1990 to 4.5 million (95% UI: 2.2-8.0) in 2019 (**Table 1**, **Figure 1B**). Over the same period, the ASDR rose from 45 (95% UI: 21.3-79.5) per 100 000 to 55.9 (95% UI: 27.2-97.6) per 100 000, with an EAPC of 0.84 (95% CI: 0.78-0.89) (**Table 1**, **Figure 1D**).

A notable gender disparity was also observed for DALYs and ASDR. In males, DALYs increased from 691.5 thousand (95% UI: 288.5-1292.4) in 1990 to 2.0 million (95% UI: 0.9-3.7) in 2019. Over the same timeframe, female DALYs rose from 1.0 million (95% UI: 0.5-1.8) to 2.5 million (95% UI: 1.3-4.3). The ASDR slightly increased from 40.5 per 100 000 (95% UI: 17.9-73.9) to 54.3 per 100 000 (95% UI: 25-97.6) in males, while rising at a slower pace in females from 49.1 (95% UI: 24.1-84.6) per 100 000 to 57.5 (95% UI: 28.6-98.4) per 100 000. Males demonstrated a higher EAPC (0.99; 95% CI: 0.93-1.05) than females (0.73; 95% CI: 0.66-0.79) (**Table 1**).

Across SDI regions, high SDI areas exhibited a rise in DALYs from 420.3 thousand (95% UI: 187.2-765.9) in 1990 to 879 thousand (95% UI: 410.8-1574.4) by 2019. The ASDR also rose from 40.5 (95% UI: 17.8-74.8) per 100 000 to 49.7 (95% UI: 23-91.8) per 100 000 over the same timeframe, with an EAPC of 0.63 (95% CI: 0.54-0.73). High-middle SDI regions exhibited rises in DALYs from 445.7 thousand (95% UI: 213.5-759) in 1990 to 963.1 thousand (95% UI: 481.4-1662.4) in 2019, and in ASDRs from 43 (95% UI: 21.1-72.5) per 100 000 to 47.6 (95% UI: 23.8-81.9) per 100 000, with an EAPC of 0.39 (95% CI: 0.29-0.49). Similarly, the middle, low-middle, and low SDI regions all exhibited upward trends, with the fastest ASDR increase observed in low-middle SDI regions (EAPC: 1.19; 95% CI: 1.09-1.29) (**Table 1**, **Figure 1D**). Among GBD regions, the highest number of DALYs in 2019 occurred in South Asia with 783.8 thousand (95% UI: 374.1-1426.2), followed by North Africa and Middle East with 685 thousand (95% UI: 356.4-1111.5). The highest ASDR in 2019 shifted to Oceania (281.4 per 100 000; 95% UI: 125.3-525.6) and the Caribbean (184.1 per 100 000; 95% UI: 93.5-310.6).Considering the changes of ASDR in different regions over time, ASDR showed declining trends in regions including East Asia and High-Income Asia Pacific, while remaining relatively stable in Eastern Sub-Saharan Africa. However, ASDRs rose in other regions like Southern Latin America and Central Asia, with Central Asia having the fastest increase (EAPC: 3.18; 95% CI: 3.01-3.36) (**Table 1**, **Figure 2D**).

Nationally, India recorded the highest DALYs in 1990, totaling 194 thousand (95% UI: 86.5-355.8), while in 2019, India and China emerged as the leading countries in DALYs with 576.3 and 452.1 thousand, respectively. Trinidad and Tobago had the highest ASDR in 1990, at 447.2 (95% CI: 221.3-746) per 100 000, with Fiji leading in 2019, at 672.7 (95% CI: 317.8-1188.4) per 100 000. Guatemala and Mongolia consistently showed the lowest ASDR, recording 4.9 in 1990 and 11.4 in 2019 per 100 000. There were increases in ASDR trends in 157 countries and territories from 1990 to 2019, while 21 countries and territories exhibited downward trends in ASDR (**Figures 2C, D**; **Supplementary Table 1**).

In 2019, the number of DALYs for type 2 diabetes due to physical inactivity in different age groups reflected the observed pattern in mortality rates, peaking in the 65-69 age group (**Figure 1F**). In high and high-middle SD) regions, DALY rates consistently rose with increasing age. In contrast, in middle, low-middle, and low SDI regions, these rates initially surged until the age group of 80-84, followed by a slight variation in individuals aged over 85 (**Figure 1H**; **Supplementary Figure 2A**). The trend in the EAPC of DALY rates across different ages paralleled the EAPC trends seen in mortality rates (**Supplementary Figure 2B**).

### Global Burden of Type 2 diabetes associated with physical inactivity by sex and age in 2019

**Figure 3:**
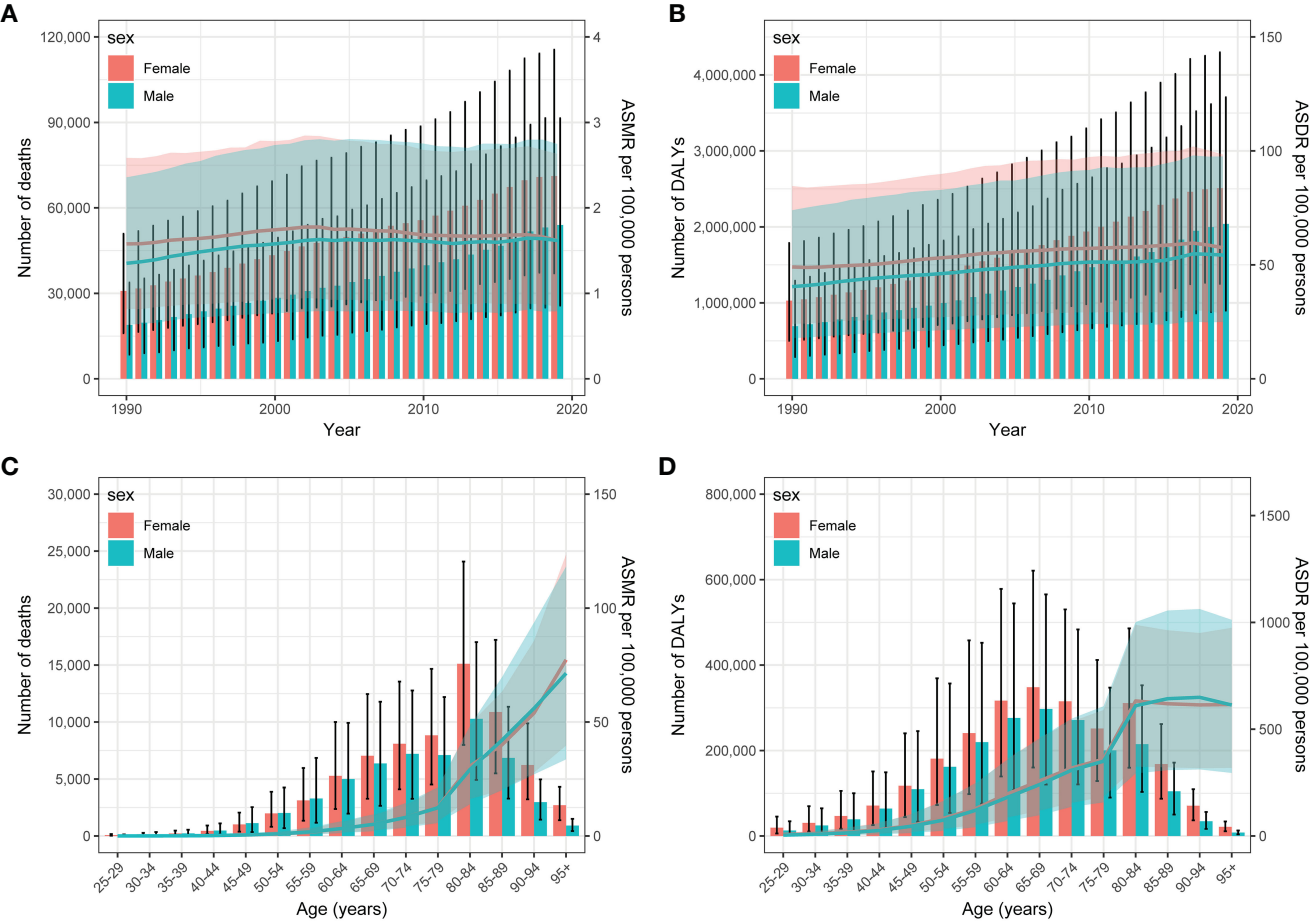
Year-specific numbers and rates of deaths **(A)** and DALYs **(C)**, and Age-specific numbers and rates of deaths **(B)** and DALYs **(D)** of type 2 diabetes attributable to physical inactivity by sex, in 2019. DALYs, disability-adjusted life years.

Between 1990 and 2019, the global numbers of deaths and DALYs from type 2 diabetes associated with physical inactivity was higher among females than males (**Figures 3A, B**). While the ASMR was consistently greater in females, this disparity between male and female ASMR narrowed over the 30-year period. The 2019 data on deaths and DALYs in different age groups due to type 2 diabetes related to physical inactivity are depicted in **Figures 3C, D**. Similarly, the death and DALY counts were higher in females. Generally, the death rate for type 2 diabetes due to physical inactivity showed an upward trend for both sexes, with the DALY rate significantly increasing in the 75-84 age bracket, but stabilizing after 85 years of age.

### Association between ASMR, ASDR of Type 2 diabetes and SDI values

**Figure 4:**
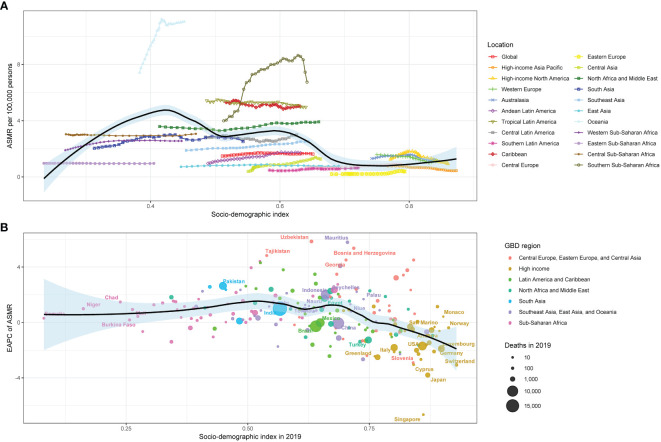
The relationship between type 2 diabetes **(A)** ASMR and SDI in 2019 by GBD region, **(B)** EAPC in ASMR and SDI in 2019 by Super GBD region. ASMR, age-standardized mortality rate; SDI, Socio-demographic Index; GBD, Global Burden of Disease Study; EAPC, estimated annual percentage change.

**Figure 5:**
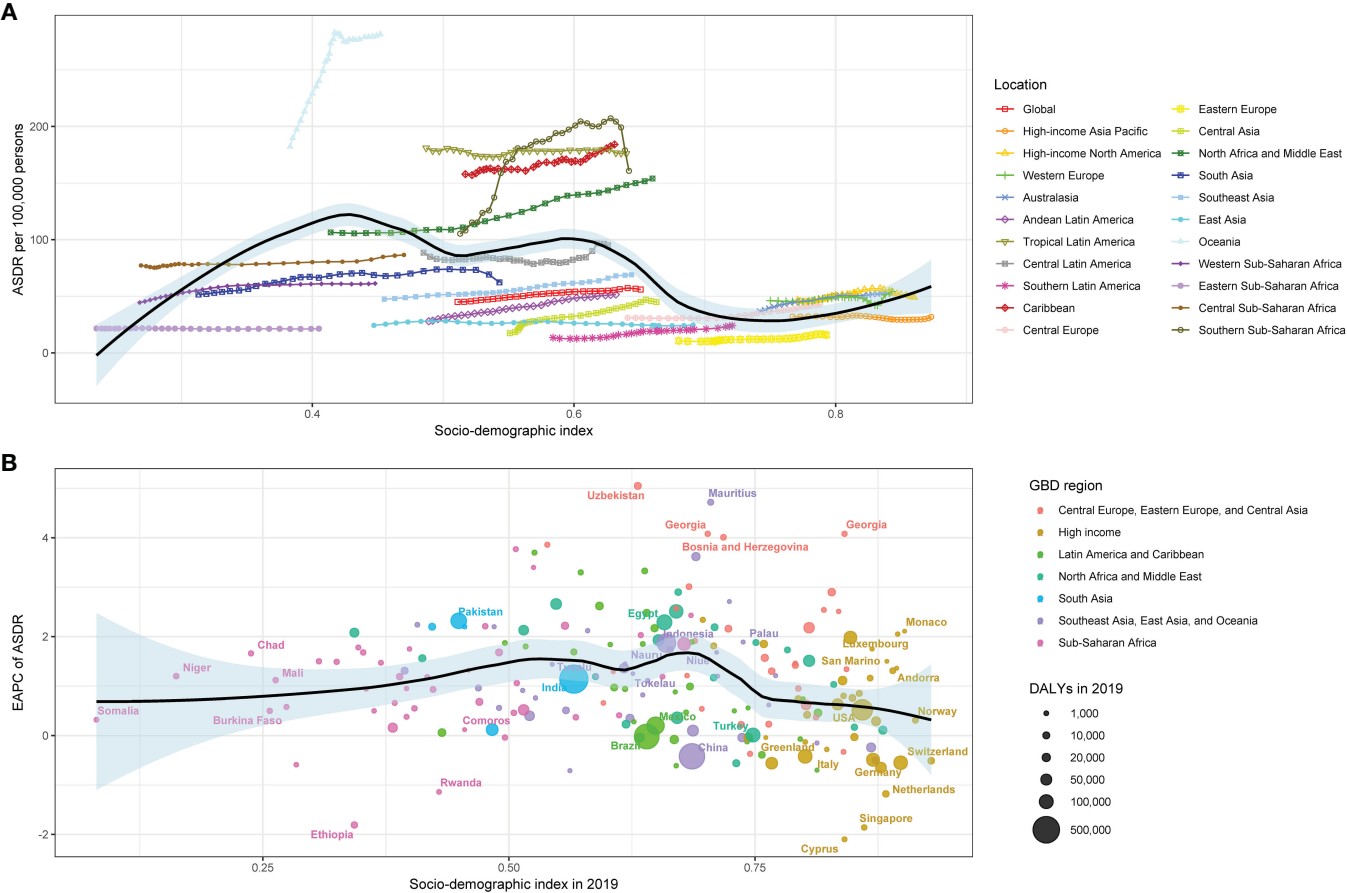
The relationship between type 2 diabetes **(A)** ASDR and SDI in 2019 by GBD region, **(B)** EAPC in ASDR and SDI in 2019 by Super GBD region. ASDR, age-standardized disability-adjusted life-years rate; SDI, Socio-demographic Index; GBD, Global Burden of Disease Study; EAPC, estimated annual percentage change.

The relationship between the ASMR for type 2 diabetes due to physical inactivity and the SDI values in various GBD regions formed an M-shape. The ASMR initially rose until reaching an SDI of around 0.43, after which it declined as SDI values increased. In 2019, the EAPC in ASMR for type 2 diabetes due to physical inactivity showed a slight negative correlation with SDI, especially when SDI exceeded 0.65. Over the span from 1990 to 2019, more than half of the 204 countries analyzed displayed an upward ASMR trend (EAPC and 95% CI above 0) (**Figure 4**). A similar pattern was noted in the relationship between the age-standardized DALY rate, SDI values, and EAPC in the age-standardized DALY rate (**Figure 5**).

## Discussion

In our research, we examined the spatial and temporal patterns of deaths and DALYs associated with physical inactivity and their influence on type 2 diabetes over the period from 1990 to 2019 in 204 countries. Our findings reveal a significant global increase in both deaths and DALYs attributable to type 2 diabetes related to physical inactivity, with deaths rising from 49.8 thousand to 125.2 thousand and DALYs more than doubling from 1.7 million to 4.5 million. While the ASMR showed a slight increase globally, notable geographical and socio-demographic disparities were observed. High SDI regions experienced a decrease in ASMR, contrasting with the stable or increasing rates in high-middle and low SDI regions. The rise in DALYs and disability rates was also more significant for males than females. The majority of deaths and DALYs attributable to physical inactivity occurred in those aged 60-89 years, predominantly in middle and low-middle SDI regions. These findings highlight the growing burden of type 2 diabetes due to insufficient physical activity across the globe, with an exceptionally high impact on elders and those in low to middle-income regions the urgent need for comprehensive public health strategies.

Type 2 diabetes, resulting from a progressive, non-autoimmune reduction in β-cell insulin secretion, often amid insulin resistance and metabolic syndrome, represents 90-95% of all diabetes cases. While the precise etiologies are unclear, type 2 diabetes does not involve the autoimmune destruction of β-cells or other known causes of diabetes (11). The complex interrelationship between physical inactivity and type 2 diabetes is rooted in intricate physiological mechanisms. Physical activity prevents type 2 diabetes through several physiological pathways (20–22). It can increase skeletal muscle glucose uptake to lower blood sugar levels. It also stimulates the release of adiponectin from adipose tissue, improving insulin sensitivity and mitigating insulin resistance. Regular physical activity aids weight control, reduces abdominal adiposity, and enhances insulin sensitivity, reducing type 2 diabetes risk (10). Activities of moderate intensity are categorized as having a MET value between 3 and 5.9, while those of vigorous intensity are characterized by a MET value of 6 or higher (23). Activities like walking briskly or raking the yard fall into the moderate-intensity category. On the other hand, vigorous intensity activities include actions such as jogging or running or lugging heavy groceries. Exercise guidelines suggests aiming for at least 150 minutes per week of aerobic exercise at a moderate-to-vigorous level, distributed across no less than three days weekly for patients with type 2 diabetes (24). Moreover, type 2 diabetes prevention efficacy varies by exercise intensity, with studies showing moderate-intensity aerobic activity reduces type 2 diabetes incidence significantly more than lower-intensity exercise (25).

Among type 2 diabetes patients, different age-group mortality and DALYs attributable to physical inactivity exhibited pronounced increases with advancing age, peaking at 80-84 and 65-69 years, respectively. This trend signifies escalated vulnerability to the deleterious impacts of physical inactivity on type 2 diabetes in older populations. The exponential rise in different age-group mortality observed in type 2 diabetes patients, mainly those aged 80-84 years, due to physical inactivity can be attributed to an interplay of factors associated with aging. Advancing age leads to declines in skeletal muscle mass and quality, impairing glucose uptake capacity (26). Additionally, increased adiposity and upregulated proinflammatory cytokines from adipose tissue inhibit insulin signaling (27, 28). Deteriorating β-cell function also reduces insulin secretion with age (29). Collectively, these changes lead to decreased insulin sensitivity, which, if not compensated by physical activity, can precipitate hyperglycemia. Moreover, common age-related comorbidities like hypertension and atherosclerosis exacerbate insulin resistance and escalate mortality risk (30). Reduced mobility in elderly individuals limits their ability to perform aerobic exercise. Thus, elderly type 2 diabetes patients rely more heavily on regular physical activity to maintain glucose homeostasis than their younger counterparts. Insufficient activity may deteriorate glycemic control, increase complications, and heighten mortality risk (10). This highlights the importance of developing physical activity programs and glucose control measures tailored to cognitive and physical limitations in elderly individuals.

over the past 30 years, the burden of type 2 diabetes due to physical inactivity has saw a notable rise in both genders, with a more marked increase in men. Furthermore, although women have a greater burden of type 2 diabetes due to physical inactivity than men, the gap in ASMR and ASDR between the genders has recently been narrowing or reversing. The underlying reasons for this trend remain largely unexplored. Possible reasons include physiological differences between sexes, such as the impact of gestational diabetes in women (31), changes in estrogen levels, and patterns of fat distribution (32). Additionally, research has found that the prognosis for women with type 2 diabetes is worse than for men due to factors such as later diagnosis of type 2 diabetes in women, increased risk of cardiovascular disease (CVD) post-menopause, and deterioration of renal function in women (33). Societal norms and roles also impact physical activity levels. Men often engage more in physical labor or recreational sports, while women may have less time for such activities due to domestic responsibilities and caregiving roles. Research indicates unpaid domestic work and family duties can engender competing demands and chronic stress in women (34). Additionally, women generally have a higher intake of sugar, and diets with a high glycemic index tend to augment abdominal fat, particularly in sedentary women, in comparison to men (32). Similarly, a study on the burden of type 2 diabetes due to high body mass index (BMI) in China found a similar disease burden pattern (30), suggesting a synergistic effect of physical inactivity and high BMI in contributing to type 2 diabetes. Given the variations in disease prevalence, societal norms, and intervention responses, a gender-specific strategy is essential in public health measures aimed at reducing the impact of physical inactivity. This approach should focus on lessening the burden of type 2 diabetes in women and tackle the rising ASMR among men.

The SDI, encompassing per capita income, education levels, and fertility rates, plays a critical role in understanding the global and regional dynamics of type 2 diabetes linked to physical inactivity. Our study highlights a complex interplay between the SDI and the impact of type 2 diabetes due to physical inactivity. In many countries with medium and low-middle SDI, physical inactivity has led to a substantial disease burden with an increasing trend. Recognizing its status as the fourth most significant risk factor for numerous NCDs and premature death, the World Health Organization aims to decrease the rate of insufficient physical activity by 10% by 2025 to enhance NCD prevention and treatment (14). Numerous studies have shown that physical inactivity is notably higher among individuals with lower income and education levels (35). Workers with only primary education or no education were found to have approximately eight times less physical activity compared to their highly educated counterparts (36). This disparity may be attributed to individuals of higher socioeconomic status engaging more in leisure-time physical activities, whereas those from lower socioeconomic backgrounds often lack the resources for such activities or opportunities to be active in other areas (37). Implementing strategies like mass media exercise promotions, community and workplace support for physical activity, and enhancing infrastructures for exercise can boost activity levels (38). This suggests that local authorities, particularly in low SDI nations, should emulate high SDI countries in intensifying efforts to promote sports and improve lifestyle choices.

This study’s inherent constraints stem from being a secondary analysis of the 2019 GBD data. Firstly, general limitations of the GBD study, including potential biases, are unavoidable and may lead to deviations from real-world figures. However, robust statistical methods were applied in the 2019 GBD study to address this issue. Secondly, GBD data are updated slowly, with the latest estimates only up to 2019, which may not capture the most current disease trends. Finally, estimating the type 2 diabetes burden attributable to physical inactivity did not account for complications arising from type 2 diabetes.

## Conclusions

This study first delineated the burden and trends of type 2 diabetes attributable to physical inactivity. Low physical activity has inflicted a substantial disease burden on the global population, predominantly in middle-aged and elderly groups. These results underscore the importance of identifying physical inactivity as a modifiable risk factor for type 2 diabetes. They provide crucial guidance for crafting impactful public health strategies and policies to counteract the escalating mortality toll of this condition. Meantime, additional research is imperative, especially in light of the aging global population, to understand the reasons behind variations in type 2 diabetes across different regions and between genders.

## Data availability statement

The original contributions presented in the study are included in the article/**Supplementary Material**. Further inquiries can be directed to the corresponding author.

## Author contributions

XY: Conceptualization, Data curation, Investigation, Methodology, Software, Supervision, Writing – original draft, Writing – review & editing. JS: Formal analysis, Project administration, Validation, Writing – original draft. WZ: Conceptualization, Resources, Visualization, Writing – original draft, Writing – review & editing.

## References

[1] Lee BX Kjaerulf F Turner S Cohen L Donnelly PD Muggah R. Transforming our world: implementing the 2030 agenda through sustainable development goal indicators. J Public Health Policy (2016) 37 Suppl 1:13–31. doi: 10.1057/s41271-016-0002-7 27638240

[2] Sun H Saeedi P Karuranga S Pinkepank M Ogurtsova K Duncan BB. IDF Diabetes Atlas: Global, regional and country-level diabetes prevalence estimates for 2021 and projections for 2045. Diabetes Res Clin Pract (2022) 183:109119. doi: 10.1016/j.diabres.2021.109119 34879977 PMC11057359

[3] Baena-Díez JM Peñafiel J Subirana I Ramos R Elosua R Marín-Ibañez A. Risk of cause-specific death in individuals with diabetes: A competing risks analysis. Diabetes Care (2016) 39(11):1987–95. doi: 10.2337/dc16-0614 27493134

[4] Safiri S Karamzad N Kaufman JS Bell AW Nejadghaderi SA Sullman MJM. Prevalence, deaths and disability-adjusted-life-years (DALYs) due to type 2 diabetes and its attributable risk factors in 204 countries and territories, 1990-2019: results from the global burden of disease study 2019. Front Endocrinol (Lausanne) (2022) 13:838027. doi: 10.3389/fendo.2022.838027 35282442 PMC8915203

[5] Al-Mawali A. Non-communicable diseases: shining a light on cardiovascular disease, Oman’s biggest killer. Oman Med J (2015) 30(4):227–8. doi: 10.5001/omj.2015.47 PMC4561645 26366254

[6] Carbone S Del Buono MG Ozemek C Lavie CJ. Obesity, risk of diabetes and role of physical activity, exercise training and cardiorespiratory fitness. Prog Cardiovasc Dis (2019) 62(4):327–33. doi: 10.1016/j.pcad.2019.08.004 31442513

[7] Guthold R Stevens GA Riley LM Bull FC. Worldwide trends in insufficient physical activity from 2001 to 2016: a pooled analysis of 358 population-based surveys with 1.9 million participants. Lancet Glob Health (2018) 6(10):e1077–86. doi: 10.1016/S2214-109X(18)30357-7 30193830

[8] Lee IM Shiroma EJ Lobelo F Puska P Blair SN Katzmarzyk PT. Effect of physical inactivity on major non-communicable diseases worldwide: an analysis of burden of disease and life expectancy. Lancet (2012) 380(9838):219–29. doi: 10.1016/S0140-6736(12)61031-9 PMC3645500 22818936

[9] Ding D Lawson KD Kolbe-Alexander TL Finkelstein EA Katzmarzyk PT van Mechelen W. The economic burden of physical inactivity: a global analysis of major non-communicable diseases. Lancet (2016) 388(10051):1311–24. doi: 10.1016/S0140-6736(16)30383-X 27475266

[10] ElSayed NA Aleppo G Aroda VR Bannuru RR Brown FM Bruemmer D. 8. Obesity and weight management for the prevention and treatment of type 2 diabetes: standards of care in diabetes-2023. Diabetes Care (2023) 46(Suppl 1):S128–39. doi: 10.2337/dc23-S008 PMC9810466 36507637

[11] ElSayed NA Aleppo G Aroda VR Bannuru RR Brown FM Bruemmer D. 2. Classification and diagnosis of diabetes: standards of care in diabetes-2023. Diabetes Care (2023) 46(Suppl 1):S19–40. doi: 10.2337/dc23-S002

[12] Diseases GBD Injuries C. Global burden of 369 diseases and injuries in 204 countries and territories, 1990-2019: a systematic analysis for the Global Burden of Disease Study 2019. Lancet (2020) 396(10258):1204–22. doi: 10.1016/S0140-6736(20)30925-9 PMC7567026 33069326

[13] Collaborators GBDD. Global age-sex-specific fertility, mortality, healthy life expectancy (HALE), and population estimates in 204 countries and territories, 1950-2019: a comprehensive demographic analysis for the Global Burden of Disease Study 2019. Lancet (2020) 396(10258):1160–203. doi: 10.1016/S0140-6736(20)30977-6 PMC7566045 33069325

[14] Xu YY Xie J Yin H Yang FF Ma CM Yang BY. The Global Burden of Disease attributable to low physical activity and its trends from 1990 to 2019: An analysis of the Global Burden of Disease study. Front Public Health (2022) 10:1018866. doi: 10.3389/fpubh.2022.1018866 36590002 PMC9798308

[15] Collaborators GBDU-M. Global, regional, and national progress towards Sustainable Development Goal 3.2 for neonatal and child health: all-cause and cause-specific mortality findings from the Global Burden of Disease Study 2019. Lancet (2021) 398(10303):870–905. doi: 10.1016/S0140-6736(21)01207-1 34416195 PMC8429803

[16] Theodoropoulou E Stavrou NAM Karteroliotis K. Neighborhood environment, physical activity, and quality of life in adults: Intermediary effects of personal and psychosocial factors. J Sport Health Sci (2017) 6(1):96–102. doi: 10.1016/j.jshs.2016.01.021 30356576 PMC6188931

[17] Collaborators GBDRF. Global burden of 87 risk factors in 204 countries and territories, 1990-2019: a systematic analysis for the Global Burden of Disease Study 2019. Lancet (2020) 396(10258):1223–49. doi: 10.1016/S0140-6736(20)30752-2 PMC7566194 33069327

[18] Yang X Fang Y Chen H Zhang T Yin X Man J. Global, regional and national burden of anxiety disorders from 1990 to 2019: results from the Global Burden of Disease Study 2019. Epidemiol Psychiatr Sci (2021) 30:e36. doi: 10.1017/S2045796021000275 33955350 PMC8157816

[19] Deng Y Li H Wang M Li N Tian T Wu Y. Global burden of thyroid cancer from 1990 to 2017. JAMA Netw Open (2020) 3(6):e208759. doi: 10.1001/jamanetworkopen.2020.8759 32589231 PMC7320301

[20] Narendran P Solomon TP Kennedy A Chimen M Andrews RC. The time has come to test the beta cell preserving effects of exercise in patients with new onset type 1 diabetes. Diabetologia (2015) 58(1):10–8. doi: 10.1007/s00125-014-3412-8 25367458

[21] Park S Hong SM Lee JE Sung SR. Exercise improves glucose homeostasis that has been impaired by a high-fat diet by potentiating pancreatic beta-cell function and mass through IRS2 in diabetic rats. J Appl Physiol (1985) (2007) 103(5):1764–71. doi: 10.1152/japplphysiol.00434.2007 17761790

[22] Medina-Contreras JML Colado-Velazquez J 3rd Gomez-Viquez NL Mailloux-Salinas P Perez-Torres I Aranda-Fraustro A. Effects of topical capsaicin combined with moderate exercise on insulin resistance, body weight and oxidative stress in hypoestrogenic obese rats. Int J Obes (Lond) (2017) 41(5):750–8. doi: 10.1038/ijo.2017.33 28163315

[23] Piercy KL Troiano RP Ballard RM Carlson SA Fulton JE Galuska DA. The physical activity guidelines for americans. JAMA (2018) 320(19):2020–8. doi: 10.1001/jama.2018.14854 PMC9582631 30418471

[24] Mendes R Sousa N Almeida A Subtil P Guedes-Marques F Reis VM. Exercise prescription for patients with type 2 diabetes-a synthesis of international recommendations: narrative review. Br J Sports Med (2016) 50(22):1379–81. doi: 10.1136/bjsports-2015-094895 26719499

[25] Jimenez-Maldonado A Virgen-Ortiz A Melnikov V Rodriguez-Hernandez A Gamboa-Dominguez A Montero S. Effect of moderate and high intensity chronic exercise on the pancreatic islet morphometry in healthy rats: BDNF receptor participation. Islets (2017) 9(1):1–10. doi: 10.1080/19382014.2016.1260796 27922332 PMC5270655

[26] Park MH Kim DH Lee EK Kim ND Im DS Lee J. Age-related inflammation and insulin resistance: a review of their intricate interdependency. Arch Pharm Res (2014) 37(12):1507–14. doi: 10.1007/s12272-014-0474-6 PMC4246128 25239110

[27] Paula FMM Leite NC Borck PC Freitas-Dias R Cnop M Chacon-Mikahil MPT. Exercise training protects human and rodent beta cells against endoplasmic reticulum stress and apoptosis. FASEB J (2018) 32(3):1524–36. doi: 10.1096/fj.201700710R 29133342

[28] Sharif K Watad A Bragazzi NL Lichtbroun M Amital H Shoenfeld Y. Physical activity and autoimmune diseases: Get moving and manage the disease. Autoimmun Rev (2018) 17(1):53–72. doi: 10.1016/j.autrev.2017.11.010 29108826

[29] Kurauti MA Soares GM Marmentini C Bronczek GA Branco RCS Boschero AC. Insulin and aging. Vitam Horm (2021) 115:185–219. doi: 10.1016/bs.vh.2020.12.010 33706949

[30] Wu Y Qin G Wang G Liu L Chen B Guan Q. Physical activity, sedentary behavior, and the risk of cardiovascular disease in type 2 diabetes mellitus patients: the MIDiab study. Engineering (2023) 20:26–35. doi: 10.1016/j.eng.2022.05.013

[31] Kim C Newton KM Knopp RH. Gestational Diabetes and the Incidence of Type 2 Diabetes: A systematic review. Diabetes Care (2002) 25(10):1862–8. doi: 10.2337/diacare.25.10.1862 12351492

[32] Kautzky-Willer A Harreiter J Pacini G. Sex and gender differences in risk, pathophysiology and complications of type 2 diabetes mellitus. Endocr Rev (2016) 37(3):278–316. doi: 10.1210/er.2015-1137 PMC4890267 27159875

[33] Stedman M Whyte MB Laing I Fryer AA Torres BM Robinson A. Failure to control conventional cardiovascular risk factors in women with type 2 diabetes might explain worse mortality. Diabetes Metab Res Rev (2023) 39(8):e3695. doi: 10.1002/dmrr.3695 37592876

[34] Gisinger T Azizi Z Alipour P Harreiter J Raparelli V Kublickiene K. Sex and gender aspects in diabetes mellitus: Focus on access to health care and cardiovascular outcomes. Front Public Health (2023) 11:1090541. doi: 10.3389/fpubh.2023.1090541 36817907 PMC9932273

[35] Gupta R Deedwania PC Sharma K Gupta A Guptha S Achari V. Association of educational, occupational and socioeconomic status with cardiovascular risk factors in Asian Indians: a cross-sectional study. PloS One (2012) 7(8):e44098. doi: 10.1371/journal.pone.0044098 22952886 PMC3430674

[36] Reddy KS Prabhakaran D Jeemon P Thankappan KR Joshi P Chaturvedi V. Educational status and cardiovascular risk profile in Indians. Proc Natl Acad Sci U.S.A (2007) 104(41):16263–8. doi: 10.1073/pnas.0700933104 PMC2042195 17923677

[37] Stalsberg R Pedersen AV. Are differences in physical activity across socioeconomic groups associated with choice of physical activity variables to report? Int J Environ Res Public Health (2018) 15(5). doi: 10.3390/ijerph15050922 PMC5981961 29734745

[38] Heath GW Parra DC Sarmiento OL Andersen LB Owen N Goenka S. Evidence-based intervention in physical activity: lessons from around the world. Lancet (2012) 380(9838):272–81. doi: 10.1016/S0140-6736(12)60816-2 PMC4978123 22818939

