# Supplementary material for: Global trends in burden of type 2 diabetes attributable to physical inactivity across 204 countries and territories, 1990-2019
Source: Front Endocrinol (Lausanne). 2024 Feb 26;15:1343002. doi: 10.3389/fendo.2024.1343002 (PMC10925666; doi:10.3389/fendo.2024.1343002)
Supplement: Supplementary file 1 [file Table_1.docx]

# Supplementary Figure legends

Supplementary Figure 1: The age distribution of (A) age-specific mortality rate and (B) EAPC in age-specific mortality rate attributable to physical inactivity by SDI region from 1990 to 2019.
EAPC, estimated annual percentage change; SDI, Socio-demographic Index.

Supplementary Figure 2: The age distribution of (A) age-specific DALYs rate and (B) EAPC in age-specific DALYs rate attributable to physical inactivity by SDI region from 1990 to 2019.
DALYs, disability-adjusted life year; EAPC, estimated annual percentage change; SDI, Socio-demographic Index.

# Supplementary Tables

Supplementary Table 1. Type 2 diabetes mellitus burden attributable to physical inactivity in 1990 and 2019 and its temporal trends from 1990 to 2019 by nation.

| **characteristic** | **1990** | | | |  | **2019** | | | |  | **EAPC (1990-2019)** | |
| --- | --- | --- | --- | --- | --- | --- | --- | --- | --- | --- | --- | --- |
|  | **Death cases, n (95% UI)** | **ASMR per 105, n (95% UI)** | **DALYs, n (95% UI)** | **ASDR per 105, n (95% UI)** |  | **Death cases, n (95% UI)** | **ASMR per 105, n (95% UI)** | **DALYs, n (95% UI)** | **ASDR per 105, n (95% UI)** |  | **ASMR, n (95% CI)** | **ASDR, n (95% CI)** |
| Afghanistan | 238.2 (109.9-449) | 3.8 (1.9-6.9) | 8733.6 (3955.9-16457.6) | 122.6 (57.5-225.1) |  | 567.7 (251.7-1104.1) | 5.7 (2.7-10.6) | 26157.8 (11500.1-49551.5) | 203.6 (97.3-364) |  | 1.82 (1.64-2) | 2.08 (1.96-2.2) |
| Albania | 3.8 (1.8-7.1) | 0.2 (0.1-0.4) | 212.3 (92.1-426.5) | 10.9 (4.9-21.4) |  | 9.3 (4.3-17.5) | 0.2 (0.1-0.4) | 693.3 (289.6-1370.4) | 16.4 (6.8-32.5) |  | -0.42 (-0.84-0) | 1.21 (1.07-1.36) |
| Algeria | 217.8 (110.3-373) | 2.7 (1.4-4.5) | 10129.6 (4877.7-17761.4) | 88.7 (45.2-152.2) |  | 802.2 (430.9-1276.2) | 3.1 (1.7-4.8) | 47846.8 (24415.1-81417.6) | 142.1 (74.3-238.9) |  | 0.93 (0.64-1.22) | 1.93 (1.74-2.13) |
| American Samoa | 1.7 (0.9-2.9) | 9.8 (5.1-15.8) | 67.2 (30.6-119.3) | 289.8 (140.4-499.6) | | 5.5 (2.9-9) | 13.1 (7-21) | 216.1 (101.6-383.6) | 443.1 (216.7-774) |  | 0.79 (0.41-1.17) | 1.2 (0.8-1.6) |
| Andorra | 0.4 (0.2-0.7) | 0.9 (0.4-1.6) | 15.6 (6.5-29.8) | 30 (12.9-56.3) |  | 1.2 (0.6-2) | 0.8 (0.4-1.3) | 62.7 (28.7-116) | 45 (20.4-83.8) |  | -0.62 (-0.68--0.55) | 1.36 (1.34-1.38) |
| Angola | 76.2 (30.8-153.7) | 2.7 (1.1-5.1) | 2722.9 (1019-5673.9) | 72.1 (29.8-141.9) |  | 248.5 (103.6-469.1) | 3.2 (1.4-5.9) | 9490.7 (3649.3-18996.3) | 87.8 (36.2-164.4) |  | 0.62 (0.58-0.65) | 0.68 (0.64-0.73) |
| Antigua and Barbuda | 4 (2.1-6.4) | 7.1 (3.7-11.3) | 96.4 (47.2-159.9) | 179.5 (84.9-305.4) |  | 7.4 (4-11.6) | 8.3 (4.6-12.9) | 229.2 (110.3-390.5) | 230.8 (114.1-381.5) | | 0.18 (0-0.36) | 0.56 (0.43-0.68) |
| Argentina | 83.7 (27.9-191.6) | 0.3 (0.1-0.7) | 2203.7 (777.2-5334.2) | 7 (2.5-17.1) |  | 176.5 (66.9-372.4) | 0.3 (0.1-0.7) | 5250.7 (2002.4-11715.8) | 9.7 (3.7-21.7) |  | 0.92 (0.39-1.46) | 1.81 (1.31-2.3) |
| Armenia | 17.7 (7.9-33.1) | 0.8 (0.3-1.4) | 592.8 (253-1164.4) | 23.3 (10.3-44.5) |  | 68.5 (31.5-127.7) | 1.7 (0.8-3.1) | 1991.4 (887.9-3868) | 47.4 (21.2-91.8) |  | 1.86 (1.18-2.54) | 1.91 (1.42-2.4) |
| Australia | 254 (127.2-408.6) | 1.4 (0.7-2.2) | 7313.6 (3354.1-12711.7) | 37.9 (17.3-66) |  | 617.3 (332-959.9) | 1.3 (0.7-2) | 22248.4 (11010.6-37412.7) | 54.4 (26.3-94.3) |  | -0.49 (-0.84--0.13) | 1.11 (0.91-1.3) |
| Austria | 178.5 (90.6-286.5) | 1.4 (0.7-2.3) | 4361.8 (2121.8-7349.7) | 35.7 (17-61.1) |  | 296.2 (149.4-468.4) | 1.4 (0.7-2.2) | 8635 (4120.9-15039.8) | 47.8 (22.4-85.5) |  | 0.05 (-0.42-0.51) | 0.76 (0.37-1.15) |
| Azerbaijan | 20.1 (9-40.5) | 0.4 (0.2-0.9) | 829.9 (346.3-1716.3) | 17.1 (7.3-33.8) |  | 92.3 (41.3-175.1) | 1.3 (0.6-2.4) | 3755.1 (1524.7-7709.1) | 42.6 (18.4-80.9) |  | 3.48 (3.08-3.87) | 3.01 (2.76-3.25) |
| Bahamas | 8.6 (4.6-14.1) | 6.4 (3.4-10.1) | 269.3 (124.1-468.1) | 177.2 (84.8-298.7) |  | 18.5 (9.8-30.4) | 5.4 (2.9-8.5) | 742.2 (344.2-1297.1) | 189.8 (92.5-325.7) |  | -1.05 (-1.26--0.85) | -0.07 (-0.2-0.06) |
| Bahrain | 14.5 (7.9-22.7) | 12.9 (7.4-19.1) | 527.1 (271.4-847.4) | 318 (174.2-487.6) |  | 114.1 (61.6-179.4) | 22.1 (12.4-32.9) | 4939.4 (2560-7998.5) | 528 (291.5-807.8) |  | 2.28 (1.77-2.8) | 1.88 (1.53-2.23) |
| Bangladesh | 764.8 (369.3-1320) | 2.3 (1.1-3.9) | 18318.6 (8225.2-33875.2) | 46.3 (21.3-82.5) |  | 2673.6 (1273.3-4545.4) | 2.8 (1.3-4.7) | 62336.2 (28864.1-116177.6) | 54.2 (25.4-96.2) |  | 0.1 (-0.63-0.84) | 0.12 (-0.43-0.68) |
| Barbados | 31.1 (17.4-47.3) | 10 (5.5-15.2) | 708.6 (378.9-1116.4) | 241.4 (123.8-387.9) | | 47.3 (26.6-71.8) | 9.6 (5.4-14.5) | 1252 (687.6-2002.9) | 258.3 (138.3-423.8) | | -0.54 (-0.74--0.33) | -0.12 (-0.25-0.02) |
| Belarus | 26.8 (13-47.7) | 0.2 (0.1-0.4) | 1450.2 (652.9-2776.8) | 11.3 (5.1-21.8) |  | 24.8 (11.8-44) | 0.1 (0.1-0.3) | 1889.7 (847-3705.8) | 11.6 (5.1-22.9) |  | -2.4 (-2.77--2.03) | -0.37 (-0.54--0.2) |
| Belgium | 238.2 (124.7-372.1) | 1.5 (0.8-2.4) | 6904 (3383.2-11924.9) | 44.8 (21.5-78.5) |  | 252.8 (133-388.4) | 0.9 (0.5-1.4) | 11724.5 (5696.1-21164) | 53.5 (24.4-99.7) |  | -2.28 (-2.54--2.02) | -0.03 (-0.4-0.34) |
| Belize | 4.8 (2.5-7.8) | 5.5 (2.9-8.8) | 124.5 (58-211.2) | 135.8 (64.2-228) |  | 19.4 (10.3-31.2) | 7.8 (4.2-12.2) | 640.3 (304.9-1099.3) | 228.4 (112.9-387.2) | | 1.39 (0.73-2.05) | 1.84 (1.28-2.41) |
| Benin | 28 (11.7-53.8) | 1.7 (0.7-3.1) | 769.3 (311.7-1496.6) | 41.2 (17-78.6) |  | 93.4 (40.2-181.8) | 2.5 (1.1-4.7) | 2958.6 (1196.6-5915.8) | 66.2 (28.1-125.6) |  | 1.49 (1.19-1.78) | 1.68 (1.44-1.92) |
| Bermuda | 2.6 (1.4-3.9) | 4.5 (2.4-6.8) | 73 (36.2-120.3) | 118.5 (59.5-193.5) |  | 3.6 (2-5.6) | 2.6 (1.4-4.1) | 131.7 (67.4-219.6) | 104.3 (52.3-178.5) |  | -2.16 (-2.26--2.07) | -0.7 (-0.81--0.6) |
| Bhutan | 3.4 (1.6-6.1) | 2.1 (1-3.6) | 112.7 (49-208.6) | 53.6 (25.3-94.5) |  | 17.1 (8.7-28.7) | 3.8 (2-6.3) | 473.4 (225-826.8) | 91.4 (44.6-156.6) |  | 2.36 (2.29-2.43) | 2.2 (2.1-2.3) |
| Bolivia (Plurinational State of) | 43.2 (15-89.2) | 1.6 (0.6-3.3) | 1225.6 (397.4-2702.5) | 40.8 (14.1-85.4) |  | 206.1 (80.2-400.6) | 2.8 (1.1-5.2) | 5745.7 (2128.5-11488.8) | 68.6 (26.1-135.1) |  | 1.74 (1.68-1.8) | 1.69 (1.64-1.74) |
| Bosnia and Herzegovina | 20.8 (9.2-39.9) | 0.6 (0.3-1.2) | 896.5 (368.3-1768.6) | 23.4 (10-45.3) |  | 132.5 (56.9-242) | 2.3 (1-4.1) | 3839.4 (1666.8-7250.1) | 64.2 (28.2-120.3) |  | 5.36 (4.53-6.19) | 4.01 (3.4-4.62) |
| Botswana | 18.7 (7.6-36.2) | 4.1 (1.7-7.8) | 504.8 (196-1025.2) | 93.9 (38.7-182.9) |  | 77.9 (32.4-150.1) | 7.5 (3.2-13.9) | 2194.6 (891.4-4281) | 172.9 (74.1-323.1) |  | 1.9 (1.46-2.35) | 2.03 (1.62-2.45) |
| Brazil | 4313.2 (2504.7-6263.6) | 5.5 (3.3-8) | 171050.4 (93463.7-262004.7) | 184.8 (103.8-278.8) | | 11318.4 (6780.4-16189.5) | 5 (3-7.1) | 424721.1 (246902.8-644023.6) | 178.3 (103.4-269.1) | | -0.28 (-0.35--0.22) | -0.02 (-0.07-0.04) |
| Brunei Darussalam | 7.3 (2.9-13.8) | 11.2 (4.9-19.8) | 227.2 (81.4-450) | 258.6 (103.8-489) |  | 15.2 (6.4-28) | 8.1 (3.7-13.7) | 602.3 (217.2-1181) | 214.5 (87.6-393.8) |  | -0.62 (-0.91--0.32) | -0.28 (-0.51--0.04) |
| Bulgaria | 92.7 (43.6-169.4) | 0.8 (0.4-1.4) | 3462.3 (1538.9-6603) | 28.1 (12.5-53.6) |  | 125.5 (59.3-227.1) | 0.8 (0.4-1.5) | 4836.6 (2229.5-8949.5) | 33.4 (15.2-62.7) |  | -0.6 (-0.95--0.24) | 0.23 (-0.03-0.5) |
| Burkina Faso | 35.5 (14.4-73) | 1.2 (0.5-2.3) | 1006 (399.7-2136.2) | 26.1 (10.8-54) |  | 87.4 (34.5-176.1) | 1.3 (0.5-2.6) | 2718.6 (1046.2-5725.7) | 33.3 (13.5-66.6) |  | 0.18 (-0.02-0.38) | 0.5 (0.25-0.75) |
| Burundi | 18.1 (6.8-40.5) | 1 (0.4-2.1) | 462.6 (168.1-1053.9) | 21.5 (8-48.6) |  | 25.9 (9.5-56.3) | 0.8 (0.3-1.8) | 750.2 (274.1-1736.4) | 19.2 (7-41.5) |  | -0.66 (-0.76--0.57) | -0.59 (-0.68--0.51) |
| Cabo Verde | 1.2 (0.5-2.2) | 0.5 (0.2-0.9) | 47.8 (19.6-96.1) | 20.4 (8.3-40.9) |  | 9.4 (4.2-17.1) | 2.3 (1-4.2) | 254.2 (107.5-486.8) | 61.4 (26.4-116.9) |  | 4.42 (3.66-5.2) | 3.4 (2.94-3.86) |
| Cambodia | 51.9 (19.7-106.7) | 1.6 (0.6-3.1) | 1387.7 (505.2-2905.8) | 35.4 (13.6-71.4) |  | 155.5 (60.2-308.9) | 1.7 (0.7-3.4) | 4944.8 (1842.1-10051.2) | 47.2 (18.4-93.7) |  | 0.27 (0.18-0.36) | 0.96 (0.82-1.11) |
| Cameroon | 83.3 (35.5-159.3) | 2.5 (1.1-4.6) | 2270.2 (926.5-4493.4) | 55.9 (24-107.4) |  | 345.8 (146.7-653.4) | 3.9 (1.8-7.1) | 10120.1 (3992.4-19818.3) | 92.3 (38.8-173.4) |  | 1.55 (1.24-1.86) | 1.68 (1.33-2.03) |
| Canada | 390 (180.9-674.1) | 1.2 (0.6-2.1) | 8884.6 (3863-15963.6) | 27.4 (11.9-49.1) |  | 806.1 (392.4-1335.6) | 1 (0.5-1.7) | 24791 (11161.5-46178.2) | 35.8 (15.9-67.7) |  | -1.47 (-2.11--0.82) | 0.29 (-0.02-0.61) |
| Central African Republic | 28.7 (11.4-58.3) | 3.2 (1.4-6.2) | 1006.1 (379.3-2080.2) | 87.2 (35.3-171.6) |  | 55.4 (22.2-112.3) | 3.5 (1.6-6.7) | 2161 (818.9-4552.9) | 100.8 (41.5-198.6) |  | 0.43 (0.27-0.58) | 0.58 (0.45-0.71) |
| Chad | 27.7 (11.2-56) | 1.2 (0.5-2.3) | 784.7 (306-1608.7) | 29.7 (11.7-60.1) |  | 73.7 (28.5-149.6) | 1.8 (0.7-3.5) | 2298.8 (876.6-4896.2) | 45.3 (17.8-93.3) |  | 1.47 (1.25-1.69) | 1.66 (1.5-1.82) |
| Chile | 80.5 (31.2-156.4) | 0.9 (0.4-1.7) | 3045.5 (1124.8-6250.1) | 31 (11.7-63.3) |  | 311.7 (138.2-570.4) | 1.3 (0.6-2.4) | 12706.7 (5252.2-23571.9) | 52.8 (21.8-98) |  | 1.15 (0.83-1.47) | 1.85 (1.56-2.14) |
| China | 4233 (1960.3-7644.4) | 0.7 (0.3-1.2) | 180708.7 (78758.2-335260.9) | 23.6 (10.7-42) |  | 11859.4 (5471.9-20891.6) | 0.7 (0.3-1.2) | 452070.1 (192468-861491.4) | 23.6 (10.3-44.2) |  | -0.06 (-0.24-0.12) | -0.42 (-0.58--0.26) |
| Colombia | 160 (57.2-328.5) | 1.1 (0.4-2.2) | 6902.8 (2305.8-15017.6) | 40.7 (13.8-86.4) |  | 493.5 (189.5-975.7) | 0.9 (0.3-1.8) | 25037.8 (9033.6-51765.8) | 47.2 (16.7-98.3) |  | -1.61 (-2.04--1.18) | -0.04 (-0.31-0.24) |
| Comoros | 1 (0.4-2.4) | 0.6 (0.2-1.4) | 23.9 (9.1-60) | 11.9 (4.6-29.5) |  | 2.5 (1-5.4) | 0.6 (0.2-1.4) | 58.5 (22.8-141.2) | 13.1 (5.1-30.6) |  | 0.02 (-0.1-0.14) | 0.12 (-0.01-0.25) |
| Congo | 36.2 (14.1-71.1) | 4.4 (1.9-8.4) | 1173.5 (443.3-2358) | 112.4 (45.4-217.7) |  | 90.1 (38.4-169.2) | 4.7 (2.2-8.4) | 3226.6 (1276-6276.2) | 124.8 (51.7-228) |  | 0.21 (0.13-0.29) | 0.37 (0.28-0.46) |
| Cook Islands | 1.4 (0.7-2.3) | 12.7 (6.3-21.5) | 35.8 (16-64.5) | 289.4 (133.5-504) |  | 3.3 (1.7-5.4) | 13.9 (7.3-22.4) | 88.8 (43.7-153.8) | 368 (181-635.9) |  | 0.07 (-0.18-0.33) | 0.62 (0.4-0.84) |
| Costa Rica | 7.5 (2.6-16.7) | 0.5 (0.2-1) | 318.9 (106.6-758) | 18.6 (6.4-43.5) |  | 24.8 (8.3-52.2) | 0.5 (0.2-1) | 1260.4 (426.2-2810.6) | 24.7 (8.3-55.8) |  | -0.76 (-1.25--0.28) | 0.47 (0.23-0.71) |
| C么te d'Ivoire | 58 (22.4-114.9) | 2.1 (0.9-4) | 1817.5 (675.8-3670.7) | 51 (20.4-99.7) |  | 211.1 (88.3-397.8) | 2.8 (1.2-5.4) | 6910.7 (2676.1-13961.1) | 72.2 (30-138.5) |  | 0.91 (0.59-1.22) | 1.29 (1.02-1.56) |
| Croatia | 38.5 (18.3-68) | 0.7 (0.3-1.2) | 1553.6 (687.9-2969.4) | 25 (11.1-47.5) |  | 79.8 (37.4-141.3) | 0.8 (0.4-1.4) | 3116 (1403.8-5863.9) | 35 (15.7-67.4) |  | 0.25 (-0.08-0.58) | 0.95 (0.85-1.06) |
| Cuba | 275 (146.6-433.7) | 2.8 (1.5-4.4) | 10963.9 (5191.7-19112.9) | 107.1 (50.5-186.9) |  | 290.2 (155.8-449.1) | 1.5 (0.8-2.3) | 20217.3 (9750.5-35489.9) | 109.2 (52.7-195.2) |  | -2.43 (-3.18--1.68) | -0.08 (-0.47-0.31) |
| Cyprus | 46 (19.6-78.8) | 7.6 (3.4-12.9) | 921.6 (382.6-1654.5) | 126.5 (52.6-222.8) |  | 59.6 (27.7-97.2) | 3.5 (1.6-5.6) | 1504.3 (650.4-2695.5) | 78.9 (34.5-141) |  | -3.04 (-3.19--2.9) | -2.1 (-2.26--1.95) |
| Czechia | 106.4 (51.5-190.5) | 0.8 (0.4-1.4) | 4689.9 (2111.7-8995.6) | 34.2 (15.2-65.3) |  | 293.5 (140.5-495.4) | 1.3 (0.6-2.2) | 13495.9 (6265.5-24891.2) | 65.5 (30.1-123.1) |  | 3.77 (2.9-4.65) | 2.9 (2.52-3.28) |
| Democratic People's Republic of Korea | 101.6 (43.1-207.1) | 0.9 (0.4-1.7) | 3357.6 (1329.5-7000.9) | 24.4 (10.2-47.9) |  | 238.1 (103.1-438.5) | 0.8 (0.4-1.5) | 8657.2 (3508.1-16867.9) | 28.1 (11.6-54.7) |  | -0.15 (-0.31-0.01) | 0.51 (0.43-0.59) |
| Democratic Republic of the Congo | 323.2 (130.2-651.7) | 3 (1.3-5.6) | 11097.6 (4310.8-23277.5) | 75 (30.5-146.4) |  | 722.5 (301.3-1377.3) | 2.8 (1.2-5) | 28094.5 (10844-57746.6) | 80.4 (33.4-158.4) |  | -0.4 (-0.48--0.31) | 0.16 (0.05-0.27) |
| Denmark | 81.7 (38.6-136.8) | 1 (0.4-1.6) | 2080 (919-3782.4) | 26 (11.2-48.7) |  | 166.2 (80.3-271.5) | 1.3 (0.6-2.1) | 4535.5 (2063-7995) | 40.6 (17.9-74.1) |  | 1.14 (0.58-1.71) | 1.31 (0.85-1.77) |
| Djibouti | 0.6 (0.2-1.5) | 0.8 (0.3-1.6) | 18.9 (6.6-44.5) | 16.8 (6.2-36.9) |  | 4.1 (1.6-8.8) | 1.1 (0.4-2.3) | 118.4 (43.6-261.2) | 24.1 (9.3-50.3) |  | 1.34 (1.26-1.41) | 1.32 (1.24-1.4) |
| Dominica | 6.2 (3.2-10) | 8.2 (4.2-13.3) | 143.7 (69.9-238.9) | 198.5 (92.9-337) |  | 7.8 (4.2-12.4) | 8.4 (4.6-13.5) | 222.7 (111.4-380.7) | 247.3 (123-423.3) |  | -0.15 (-0.3-0) | 0.5 (0.38-0.61) |
| Dominican Republic | 94.6 (50.4-153.3) | 2.9 (1.5-4.5) | 3269.4 (1622.2-5437.5) | 85.8 (43.9-141.1) |  | 412.8 (218.7-672.1) | 4.6 (2.5-7.5) | 15164 (7780.9-25496.1) | 158.9 (83-264.2) |  | 2.29 (2.08-2.49) | 2.62 (2.44-2.81) |
| Ecuador | 55.1 (22-108.7) | 1.2 (0.5-2.3) | 1804.7 (653.6-3676.2) | 35.1 (13.2-70) |  | 359.7 (151.7-676.2) | 2.7 (1.2-5) | 10760.6 (4310-21065.3) | 73.2 (29.9-141.2) |  | 2.8 (2.52-3.08) | 2.48 (2.28-2.67) |
| Egypt | 846.1 (458.9-1326.3) | 3.5 (1.9-5.4) | 29008.5 (14601.9-47395.6) | 99.4 (52.3-157.4) |  | 2896.7 (1506.9-4828.8) | 5.3 (2.9-8.6) | 120102.7 (61546.9-201904.4) | 181.8 (96.5-296.6) |  | 1.74 (1.63-1.85) | 2.29 (2.21-2.36) |
| El Salvador | 12.2 (4.1-27.6) | 0.4 (0.1-1) | 442.6 (152.6-1055) | 15.3 (5.3-35.9) |  | 80.3 (26.2-175.2) | 1.3 (0.4-2.8) | 2332.5 (745.9-5362.6) | 39.1 (12.5-89.8) |  | 3.8 (3.41-4.2) | 3.3 (3-3.6) |
| Equatorial Guinea | 4.4 (1.7-8.8) | 2.8 (1.2-5.5) | 148.8 (55.3-298.5) | 77 (31.2-152.5) |  | 19.3 (8.6-36.7) | 5.5 (2.5-9.8) | 646.4 (264.2-1249.2) | 136.7 (59.6-256.3) |  | 2.75 (2.6-2.89) | 2.43 (2.28-2.58) |
| Eritrea | 5.7 (2-14.2) | 0.9 (0.3-2) | 175.5 (64.6-446.4) | 20.8 (7.8-49.9) |  | 19.7 (7.3-44) | 1.2 (0.4-2.5) | 581.2 (210.8-1346) | 26.1 (9.7-56.6) |  | 1.07 (0.91-1.23) | 0.95 (0.84-1.06) |
| Estonia | 3.1 (1.5-5.6) | 0.2 (0.1-0.3) | 199.7 (89.1-383.9) | 9.8 (4.4-18.9) |  | 8.6 (4.1-15) | 0.3 (0.1-0.5) | 480.3 (214.9-914.4) | 17.5 (7.6-33.9) |  | 2.34 (1.52-3.18) | 2.51 (2.22-2.81) |
| Eswatini | 14.2 (5.9-27.6) | 6.1 (2.6-11.5) | 372.4 (146.6-721.5) | 136.9 (55.7-261.8) |  | 54.6 (23.1-102.8) | 11.7 (5.2-21.8) | 1474.7 (597.8-2777.2) | 264.6 (110.6-494.3) | | 2.43 (1.73-3.13) | 2.4 (1.71-3.09) |
| Ethiopia | 178.8 (63.7-403.8) | 1.2 (0.4-2.7) | 5268.9 (1827.3-12490.1) | 28 (10.2-62.2) |  | 262.9 (101.3-537.5) | 0.9 (0.3-1.7) | 6720.6 (2481.9-14836.2) | 18.2 (6.9-38.5) |  | -1.4 (-1.47--1.33) | -1.81 (-1.89--1.73) |
| Fiji | 42 (18.5-79.4) | 15.1 (7.3-27.3) | 1274.4 (531.6-2476.5) | 358.6 (160.8-664.7) | | 170.3 (80.1-303.2) | 29 (14.8-49.6) | 4939.5 (2210.9-9141.9) | 672.7 (317.8-1188.4) | | 1.73 (1.2-2.27) | 1.74 (1.25-2.23) |
| Finland | 52.3 (25.5-86.1) | 0.7 (0.4-1.2) | 2375.3 (1042-4491.4) | 33.6 (14.7-64.2) |  | 56.3 (27.2-91.8) | 0.4 (0.2-0.7) | 5151.6 (2224.8-9730.3) | 46.3 (18.7-91.6) |  | -2.67 (-2.98--2.35) | 0.85 (0.5-1.2) |
| France | 1036.9 (567.1-1556) | 1.2 (0.6-1.7) | 24590.6 (12651.9-40076.8) | 29.3 (14.9-48.4) |  | 1991.6 (1088.1-2999.1) | 1.1 (0.6-1.7) | 50845 (26697.3-82035.3) | 37.9 (19.2-63.8) |  | -0.32 (-0.87-0.24) | 0.63 (0.31-0.95) |
| Gabon | 17.2 (7-34.1) | 3.8 (1.6-7.3) | 484.7 (193.4-974.9) | 90.7 (37.6-178.7) |  | 42.9 (18.5-80.6) | 5.3 (2.4-9.8) | 1286.1 (522.6-2577) | 128.7 (55.5-245.4) |  | 1.11 (0.84-1.38) | 1.19 (0.94-1.43) |
| Gambia | 3.7 (1.5-7.6) | 1.5 (0.6-2.8) | 109.2 (41.6-223.5) | 34.9 (13.9-69.1) |  | 19.1 (7.8-37.7) | 2.5 (1-4.8) | 529 (212.2-1055.2) | 60 (24.6-116.8) |  | 1.68 (1.46-1.91) | 1.75 (1.56-1.94) |
| Georgia | 25.3 (11.5-47.7) | 0.4 (0.2-0.8) | 1045.7 (444-2029.8) | 17.3 (7.5-33.5) |  | 71.4 (32.1-130.8) | 1.1 (0.5-2) | 2564.8 (1127.5-5016.7) | 41.9 (18.3-82.4) |  | 4.51 (3.89-5.14) | 4.08 (3.65-4.51) |
| Germany | 2038.6 (920.4-3519.8) | 1.5 (0.7-2.6) | 54264 (22327.3-101927.3) | 41.5 (16.9-78.7) |  | 2574.4 (1202.3-4321.1) | 1.1 (0.5-1.8) | 92228.5 (39096.2-169866.1) | 48.2 (19.3-92.7) |  | -1.89 (-2.22--1.57) | -0.55 (-1.16-0.08) |
| Ghana | 78 (31.6-156.6) | 1.7 (0.7-3.4) | 2367.6 (926.6-4875.9) | 42.3 (17.1-83.8) |  | 370.2 (158.5-716.6) | 3 (1.3-5.6) | 11573.5 (4706.9-23182.5) | 77.5 (32.8-152.8) |  | 1.94 (1.61-2.28) | 2.22 (1.87-2.58) |
| Greece | 93.6 (39.6-169.3) | 0.6 (0.3-1.1) | 3198.1 (1222.1-6275.2) | 20.9 (8-40.9) |  | 132.5 (58.1-227.8) | 0.4 (0.2-0.8) | 6923.7 (2683.5-13561.5) | 29.1 (10.5-59.6) |  | -0.85 (-1.45--0.25) | 0.74 (0.12-1.36) |
| Greenland | 0.3 (0.1-0.6) | 1.3 (0.5-2.3) | 8.6 (3.2-17.1) | 28.5 (11.3-54.1) |  | 0.4 (0.2-0.8) | 0.8 (0.3-1.4) | 21.5 (8.2-43.4) | 32.5 (13-62.6) |  | -2.44 (-2.74--2.14) | -0.04 (-0.25-0.16) |
| Grenada | 6.4 (3.4-10.3) | 8.1 (4.1-13.1) | 147.4 (71.8-244.7) | 202.2 (93.1-349.5) |  | 9.2 (4.8-14.9) | 9.6 (5.1-15.3) | 283.8 (133.2-489.4) | 261.5 (129.2-438.7) | | 0.64 (0.38-0.91) | 0.88 (0.73-1.03) |
| Guam | 2.1 (1-3.7) | 4.2 (2-7) | 72.3 (30.4-131.4) | 101.7 (46.6-177.4) |  | 4.2 (2.1-7.2) | 2.3 (1.1-3.9) | 175 (78.7-324.6) | 93.9 (42.6-173.1) |  | -2.29 (-2.69--1.89) | -0.15 (-0.45-0.15) |
| Guatemala | 3.8 (1.6-10.5) | 0.1 (0.1-0.4) | 171.7 (77.8-507.6) | 4.9 (2.1-14.1) |  | 60.1 (22.3-157.1) | 0.6 (0.2-1.6) | 1801.9 (749.9-5079.3) | 16.6 (6.8-47.1) |  | 4.31 (3.49-5.13) | 3.7 (3.14-4.27) |
| Guinea | 39.4 (16.8-77.2) | 1.4 (0.6-2.7) | 1034 (431.2-2041.5) | 33.2 (14.1-65.2) |  | 94.3 (38.7-177.1) | 2.1 (0.9-3.9) | 2563.2 (1009.8-5121.1) | 50.3 (20.3-99.6) |  | 1.4 (1.19-1.61) | 1.49 (1.33-1.65) |
| Guinea-Bissau | 7.6 (2.9-15.8) | 2.4 (1-4.7) | 220.1 (82.4-468.6) | 57.9 (22.4-118.7) |  | 16.5 (6.6-32.5) | 3.2 (1.3-6.1) | 508.7 (187.6-1045) | 77.3 (31-151.3) |  | 0.93 (0.69-1.17) | 0.94 (0.72-1.15) |
| Guyana | 30.8 (14.5-52.2) | 9.4 (4.6-15.5) | 965 (421.6-1725.3) | 255.8 (117.2-443.1) | | 67.5 (33.8-116.2) | 12.4 (6.5-20.6) | 2300.1 (1054-4143.7) | 363.4 (175-633.9) |  | 0.61 (0.13-1.09) | 0.94 (0.52-1.36) |
| Haiti | 242 (110.3-441.5) | 9 (4.3-15.6) | 7344 (3093.5-13757.1) | 229.1 (104-413.5) |  | 429.6 (195.8-818.3) | 7.6 (3.5-13.7) | 15404 (6493.9-30510.9) | 224.9 (103.5-427) |  | -0.45 (-0.52--0.38) | 0.06 (-0.03-0.14) |
| Honduras | 4.6 (1.6-11.3) | 0.3 (0.1-0.6) | 278.2 (92.8-720.8) | 13.8 (4.6-34.2) |  | 27 (9.1-65.3) | 0.5 (0.2-1.3) | 1401.1 (457.9-3392.9) | 24 (8-57.9) |  | 2.62 (2.31-2.93) | 1.87 (1.75-1.99) |
| Hungary | 82.1 (39.1-147.8) | 0.6 (0.3-1) | 3256.5 (1440.1-6275.6) | 22.4 (10-42.8) |  | 142.9 (66.5-251.7) | 0.7 (0.3-1.2) | 6016.6 (2685.4-11361.9) | 31.1 (13.7-60.2) |  | 0.83 (0.43-1.23) | 1.42 (1.18-1.67) |
| Iceland | 1.9 (0.9-3) | 0.6 (0.3-1) | 74.1 (34.4-133.1) | 26 (11.9-47.3) |  | 3.4 (1.8-5.4) | 0.5 (0.3-0.8) | 233.7 (108.3-422.9) | 44 (19.7-81.4) |  | -0.87 (-1.05--0.69) | 1.75 (1.71-1.78) |
| India | 5238.9 (2483.5-9416) | 1.9 (0.9-3.2) | 194043.7 (86514.3-355788.4) | 49.6 (23.7-88.9) |  | 19070.6 (9589.6-32291.6) | 2.3 (1.1-3.7) | 576283.5 (277326.6-1049494.5) | 56.7 (28.1-100.4) |  | 0.99 (0.64-1.34) | 1.14 (0.89-1.4) |
| Indonesia | 1773.3 (709.2-3489.6) | 2.2 (0.9-4.2) | 55565.9 (20331.7-115444.1) | 58.1 (22.9-116.3) |  | 6970.4 (2927.4-13190.3) | 3.9 (1.7-7) | 216491.3 (83927.1-420312.4) | 100.6 (41.1-187) |  | 2.01 (1.9-2.13) | 1.87 (1.76-1.98) |
| Iran (Islamic Republic of) | 313.2 (160.8-505.4) | 1.8 (1-2.8) | 16198.4 (7639-28258.5) | 64.7 (32.9-108.8) |  | 2004.2 (1075.3-3073.9) | 3.2 (1.7-4.8) | 89980.1 (43932.4-151683.9) | 123.3 (61.5-202.4) |  | 2.27 (2.03-2.51) | 2.51 (2.37-2.65) |
| Iraq | 535.8 (284.7-831) | 7.7 (4.2-11.8) | 17467.8 (8892.7-28730.7) | 220.6 (117.3-355.9) | | 1423.1 (791.4-2239.9) | 7.6 (4.3-11.4) | 58592.1 (30148.4-95911.4) | 248.4 (136.7-392.4) | | -0.11 (-0.23-0.02) | 0.36 (0.29-0.43) |
| Ireland | 58.5 (33.1-87) | 1.5 (0.8-2.2) | 1518.5 (812.7-2416.7) | 37.6 (20-59.8) |  | 78.3 (44.6-117.7) | 1 (0.6-1.5) | 4124.4 (2104.6-6905.2) | 57 (29-96.7) |  | -1.62 (-1.82--1.42) | 1.16 (0.96-1.36) |
| Israel | 97.7 (48.8-157.9) | 2.2 (1.1-3.5) | 2716 (1265.8-4633) | 56.6 (26.1-96.7) |  | 374.6 (193-580.7) | 2.9 (1.5-4.5) | 9396.8 (4647.5-15905.9) | 80.7 (39.3-139) |  | -0.13 (-1.14-0.89) | 0.42 (-0.28-1.13) |
| Italy | 2423.8 (1282.2-3714.5) | 2.7 (1.4-4.1) | 64866.8 (32972.6-105274.9) | 73.2 (36.8-120.4) |  | 3092.6 (1613.1-4787.8) | 1.7 (0.9-2.6) | 95326.1 (46935.2-160682.8) | 67.8 (32.2-118) |  | -1.82 (-1.96--1.68) | -0.42 (-0.75--0.08) |
| Jamaica | 155.4 (84.6-233.9) | 8.5 (4.6-13) | 3850.7 (1978.1-6099.6) | 217.2 (109.6-347.3) | | 369.4 (202.6-576.4) | 11.7 (6.4-18.3) | 9405.2 (4956.3-15476.6) | 314.1 (166-516.4) |  | 0.86 (0.58-1.14) | 0.99 (0.76-1.22) |
| Japan | 1008.8 (459.6-1733) | 0.6 (0.3-1.1) | 47062.8 (18569.7-90507.6) | 28 (11-53.8) |  | 1024.6 (473.7-1730.3) | 0.2 (0.1-0.4) | 81577.5 (35360.8-150996.1) | 26 (9.6-52.1) |  | -3.8 (-4.26--3.33) | -0.49 (-0.72--0.25) |
| Jordan | 78.2 (40.6-129) | 8.3 (4.4-13.5) | 2173.2 (1040-3826.6) | 183.3 (93.2-308.4) |  | 274.7 (145-435.6) | 6 (3.3-9.4) | 10359.6 (4969-17791.8) | 168 (85.8-274.7) |  | -1.39 (-1.8--0.97) | -0.56 (-0.91--0.21) |
| Kazakhstan | 46.8 (22.8-83.4) | 0.4 (0.2-0.7) | 2932.2 (1261-5676.1) | 24 (10.7-45.6) |  | 179.4 (86.3-310) | 1.2 (0.6-2.1) | 8843.3 (3939.2-16978) | 53.3 (24.3-99.5) |  | 2.41 (1.59-3.23) | 2.16 (1.81-2.52) |
| Kenya | 31.9 (12.2-70.7) | 0.5 (0.2-1.1) | 829.2 (311.7-1931.8) | 11.4 (4.4-25.7) |  | 99.4 (37.7-217.7) | 0.7 (0.3-1.4) | 2893.5 (1071.6-6658.3) | 14.7 (5.5-32.5) |  | 0.8 (0.73-0.86) | 1.06 (0.98-1.13) |
| Kiribati | 4.4 (1.9-8.3) | 13.6 (6.3-24.1) | 142 (59.1-276.3) | 361.3 (158.2-677.3) | | 11.1 (4.8-20.9) | 19.9 (9.3-34.7) | 372.4 (152.5-707.6) | 514 (223.4-939.4) |  | 0.86 (0.35-1.37) | 0.76 (0.26-1.26) |
| Kuwait | 22.3 (13-32.9) | 5.1 (3.1-7.4) | 1219 (638-1993.5) | 180.6 (101-278.1) |  | 65.2 (37.5-96.8) | 3.4 (2-5) | 6358.5 (3236.6-10606.9) | 200.7 (107.5-323) |  | -1.64 (-2.31--0.97) | 0.17 (-0.26-0.6) |
| Kyrgyzstan | 7 (3.2-13.3) | 0.2 (0.1-0.5) | 337.8 (144.5-679.7) | 11.3 (4.9-22.5) |  | 12 (5.4-23.5) | 0.3 (0.1-0.6) | 727.5 (296.9-1469.6) | 16.5 (7-32.1) |  | -0.18 (-0.56-0.2) | 0.66 (0.42-0.9) |
| Lao People's Democratic Republic | 25.4 (9.5-55.6) | 1.7 (0.6-3.4) | 755.1 (274.9-1685.6) | 41.1 (15.8-87.1) |  | 62.1 (24.9-121) | 2 (0.8-3.8) | 2026.1 (762.3-4179.5) | 53.9 (21.3-109.5) |  | 0.19 (0.01-0.37) | 0.56 (0.36-0.76) |
| Latvia | 12 (5.8-20.7) | 0.3 (0.2-0.6) | 634.1 (299.3-1171) | 17.7 (8.3-33) |  | 34.3 (17.1-56) | 0.7 (0.4-1.2) | 1352 (658.4-2382) | 33.4 (15.8-60.6) |  | 3.42 (2.97-3.87) | 2.54 (2.33-2.75) |
| Lebanon | 59.3 (32.2-93.6) | 3.1 (1.7-4.9) | 2514 (1260.9-4230.7) | 112.5 (58.2-185.2) |  | 135.7 (72.8-212.8) | 2.6 (1.4-4.2) | 7974 (4134.2-13238.6) | 153.1 (79.3-253.8) |  | -0.4 (-0.65--0.14) | 1.17 (0.97-1.36) |
| Lesotho | 21.5 (8.6-42.5) | 2.6 (1.1-5) | 552.7 (217.7-1108.5) | 58.8 (23.5-116.5) |  | 67.5 (27.5-132) | 6.6 (2.8-12.6) | 1750.2 (678.2-3515.9) | 145.5 (59.6-286.5) |  | 3.95 (3.61-4.3) | 3.77 (3.47-4.08) |
| Liberia | 19.5 (8.3-36.6) | 2.2 (1-3.9) | 586.2 (242.7-1136.4) | 55.9 (24.3-106.4) |  | 44.2 (18.5-82.3) | 2.9 (1.3-5.4) | 1510.7 (606.7-2992.3) | 80.2 (33.8-152.5) |  | 1.15 (1-1.3) | 1.47 (1.32-1.62) |
| Libya | 36.8 (19.4-59.7) | 2.3 (1.2-3.6) | 1895.2 (934.6-3200) | 100.5 (51.3-165.9) |  | 134.3 (69.6-223.4) | 3 (1.6-4.9) | 9433.6 (4713.1-15878.9) | 175.9 (90.6-292.2) |  | 1.53 (1.29-1.78) | 2.19 (2.06-2.32) |
| Lithuania | 12.7 (6.2-21.4) | 0.3 (0.1-0.5) | 750.9 (349-1417.3) | 16.6 (7.6-31.3) |  | 22.3 (11.3-37.4) | 0.3 (0.2-0.6) | 1341.9 (640-2432.7) | 23.4 (10.9-43.9) |  | 0.62 (0.44-0.8) | 1.34 (1.18-1.5) |
| Luxembourg | 6.4 (3.2-10.5) | 1.2 (0.6-2) | 157.1 (71.2-279.8) | 28.8 (13.1-51.9) |  | 8.5 (4.2-13.8) | 0.7 (0.4-1.2) | 541.3 (232.9-1021.7) | 54.4 (22.5-105) |  | -1.87 (-2.01--1.72) | 2.05 (1.69-2.42) |
| Madagascar | 26.9 (10.3-59.2) | 0.7 (0.3-1.5) | 711 (273.9-1580.1) | 15.4 (6-33.2) |  | 51.1 (19.1-117.6) | 0.7 (0.3-1.6) | 1590.9 (599.8-3807.5) | 17.3 (6.6-38.4) |  | 0.14 (0.02-0.25) | 0.38 (0.3-0.46) |
| Malawi | 31 (12.4-66.5) | 1.1 (0.5-2.3) | 857.9 (325.3-1865.7) | 25.3 (10.1-53) |  | 62 (24.9-127.3) | 1.2 (0.5-2.4) | 1761.8 (695.4-3796.7) | 27.4 (11.2-55.6) |  | 0.28 (0.16-0.4) | 0.66 (0.52-0.81) |
| Malaysia | 207.1 (97.4-355) | 2.6 (1.3-4.5) | 7280.7 (3156.5-13198.8) | 82.9 (37.9-146.6) |  | 360.3 (175.5-636.8) | 1.6 (0.8-2.7) | 21968.3 (9955.8-40356.6) | 83.5 (38.8-149.1) |  | -2.66 (-3.19--2.12) | -0.04 (-0.26-0.19) |
| Maldives | 2 (0.8-3.7) | 3.1 (1.5-5.4) | 65.7 (25.9-130.6) | 81.3 (36.2-148.4) |  | 5.2 (2.5-8.7) | 2.2 (1.1-3.6) | 212 (93-388.3) | 73.4 (34.1-128.8) |  | -1.74 (-2.04--1.43) | -0.71 (-0.99--0.44) |
| Mali | 40.7 (16.8-82.1) | 1.3 (0.6-2.6) | 1150.6 (444-2365) | 30.9 (12.6-61.5) |  | 115.3 (48-227.2) | 1.8 (0.8-3.4) | 3360.6 (1293.4-6925.5) | 43.6 (17.8-87.5) |  | 0.93 (0.7-1.16) | 1.12 (0.94-1.29) |
| Malta | 15.7 (9.3-22.7) | 3.9 (2.4-5.7) | 476.5 (263.3-724.5) | 112.6 (62.3-171.3) |  | 21.2 (12.1-30.7) | 2.1 (1.2-3) | 916.2 (498-1449.2) | 107.3 (57.5-172.7) |  | -2.13 (-2.35--1.91) | -0.13 (-0.24--0.02) |
| Marshall Islands | 1.2 (0.6-2.2) | 9.3 (4.5-15.8) | 45.7 (19.9-85.7) | 273.3 (122.3-492.7) | | 3.8 (1.6-7) | 13.9 (6.6-23.8) | 168.3 (70.2-319) | 447.3 (199.9-821.4) | | 1.15 (0.84-1.47) | 1.44 (1.1-1.78) |
| Mauritania | 42.3 (22.7-68.7) | 4.9 (2.6-7.8) | 1195.8 (629.5-1971.7) | 121.6 (64.4-198.3) |  | 90.1 (48.3-144.3) | 5.1 (2.8-8.1) | 2612 (1394.5-4242.2) | 128.1 (69.3-204.5) |  | 0 (-0.14-0.14) | -0.04 (-0.16-0.08) |
| Mauritius | 25.2 (11.1-45.4) | 3.8 (1.7-6.7) | 838.2 (351.4-1569.4) | 114.9 (49.8-213.9) |  | 183.8 (86.8-315.5) | 11.2 (5.4-19) | 5057.6 (2268.6-9082.7) | 287.5 (131.6-508.2) | | 5.79 (4.75-6.84) | 4.72 (3.93-5.51) |
| Mexico | 1838.6 (749.6-3425.7) | 4.8 (2-8.8) | 63804.4 (24271.9-122962.5) | 146 (57.2-276.9) |  | 5594.5 (2311.8-10298.5) | 5.1 (2.2-9.4) | 179597 (69680-344579.8) | 153.4 (60.2-291.8) |  | -0.02 (-0.27-0.24) | 0.2 (-0.04-0.45) |
| Micronesia (Federated States of) | 4.2 (1.9-7.3) | 10.7 (5.3-18.1) | 128.7 (56.9-233.1) | 273 (124.7-481.9) |  | 11.8 (5.3-22) | 20.9 (9.9-36.2) | 389.5 (166.6-742.2) | 533.7 (247.4-970.9) | | 2.11 (1.63-2.59) | 2.12 (1.58-2.66) |
| Monaco | 0.4 (0.2-0.6) | 0.4 (0.2-0.7) | 16.6 (8-29.1) | 24.6 (11.2-44.7) |  | 0.5 (0.3-0.8) | 0.5 (0.2-0.7) | 38.3 (18.6-67.9) | 45 (20.7-83.2) |  | 0.42 (0.25-0.59) | 2.11 (2.08-2.13) |
| Mongolia | 1.8 (0.8-3.5) | 0.2 (0.1-0.4) | 77.2 (31.7-154.1) | 7.9 (3.3-15.6) |  | 4.1 (1.8-8.2) | 0.2 (0.1-0.5) | 241.8 (94.2-506.6) | 11.4 (4.8-21.9) |  | 0.37 (0.12-0.62) | 1.29 (1.23-1.36) |
| Montenegro | 4.2 (2-7.7) | 0.7 (0.4-1.3) | 185.5 (81.1-360.9) | 30.6 (13.7-58.5) |  | 9.1 (4.3-15.7) | 1 (0.5-1.7) | 448 (199.6-857.8) | 46 (20.4-87.8) |  | 1.21 (1.09-1.33) | 1.46 (1.42-1.5) |
| Morocco | 219.9 (115-357.6) | 2 (1.1-3.1) | 9839.1 (4884.8-16826.5) | 73.3 (37.4-122.1) |  | 949 (508.1-1566.2) | 3.5 (1.9-5.7) | 46452.7 (23361.9-78481.8) | 147.4 (76.9-244.7) |  | 2.39 (2.21-2.56) | 2.66 (2.56-2.76) |
| Mozambique | 35.1 (13.9-81.7) | 0.9 (0.3-1.9) | 937.9 (360.3-2208.9) | 18.1 (7.2-41.3) |  | 87.2 (32.7-195.1) | 1.1 (0.4-2.4) | 2449.5 (902-5661.5) | 25.2 (9.5-55.8) |  | 1.27 (1.15-1.39) | 1.5 (1.38-1.62) |
| Myanmar | 532.5 (207.8-1082) | 2.9 (1.2-5.7) | 14602.9 (5497.2-30427.8) | 68 (26.7-136.6) |  | 1199.7 (496.6-2247.4) | 3.3 (1.4-5.9) | 32865.9 (12809.2-64668.2) | 77.8 (32-150.4) |  | 0.32 (0.28-0.36) | 0.4 (0.35-0.45) |
| Namibia | 34.6 (16.7-60.4) | 5.4 (2.7-9.4) | 952.7 (442.4-1686.6) | 133.4 (64.3-234.2) |  | 81.1 (38.7-140.4) | 6.7 (3.2-11.4) | 2239.5 (1054.2-3988.3) | 165 (78.4-290.5) |  | 0.33 (-0.11-0.77) | 0.41 (0-0.81) |
| Nauru | 0.3 (0.1-0.6) | 11.1 (5.1-20.1) | 9.9 (3.9-20.4) | 260.2 (116.9-494.8) | | 0.5 (0.2-1) | 17.3 (8.4-32) | 19.1 (7.4-41.1) | 428.6 (195.5-821.6) | | 1.26 (1.04-1.49) | 1.45 (1.22-1.68) |
| Nepal | 51.3 (23.3-94.9) | 0.9 (0.4-1.7) | 1948.1 (831-3934.2) | 25.7 (11.7-48.1) |  | 276.2 (132.3-480.8) | 1.8 (0.9-3.2) | 9441.8 (4114.9-18005.9) | 48.7 (22.2-90) |  | 2.57 (2.31-2.84) | 2.2 (2.02-2.37) |
| Netherlands | 228.7 (86.4-440) | 1.1 (0.4-2.2) | 4842.7 (1730.4-9882.2) | 23.7 (8.4-48.2) |  | 268.7 (106.4-497.3) | 0.7 (0.3-1.3) | 7289.7 (2698.7-14962.1) | 20.8 (7.5-43.6) |  | -2.1 (-2.61--1.59) | -1.18 (-1.83--0.53) |
| New Zealand | 39.3 (17.6-68.5) | 1 (0.5-1.8) | 1193.1 (501.6-2189.5) | 31 (12.9-57.3) |  | 87.3 (43.8-139.1) | 1 (0.5-1.6) | 3332.9 (1571.8-5798.6) | 44.2 (20.3-78.7) |  | -0.64 (-1.09--0.18) | 0.8 (0.57-1.03) |
| Nicaragua | 9 (3.1-20.5) | 0.7 (0.2-1.6) | 321.4 (109.2-772.3) | 22.1 (7.7-52.7) |  | 56.5 (19.4-124.3) | 1.6 (0.6-3.5) | 1669.2 (567.1-4000.3) | 40.8 (14.2-94.8) |  | 2.44 (1.81-3.07) | 1.8 (1.49-2.11) |
| Niger | 29.9 (12-59.7) | 1.5 (0.6-2.9) | 807.2 (309.6-1697.3) | 32.7 (13.3-64.1) |  | 109.4 (45.3-212.2) | 2 (0.9-3.8) | 3235 (1258.5-6490.6) | 47 (19.7-91.8) |  | 0.99 (0.8-1.18) | 1.2 (1.09-1.32) |
| Nigeria | 685.4 (291.6-1310.8) | 2 (0.9-3.7) | 18582.8 (7505.2-36782.7) | 45.8 (19.5-89.1) |  | 1561.3 (674.1-2902.6) | 2.5 (1.1-4.5) | 43447.9 (17860.6-83903.5) | 55.5 (23.6-103.5) |  | 0.69 (0.52-0.85) | 0.52 (0.38-0.66) |
| Niue | 0.2 (0.1-0.4) | 9.3 (4.5-16.4) | 5.1 (2.4-9.2) | 232.1 (105.9-422.7) | | 0.3 (0.1-0.5) | 14.4 (7-25.7) | 8.5 (4-15.5) | 398 (184.4-718.1) |  | 1.31 (1-1.61) | 1.68 (1.4-1.97) |
| North Macedonia | 17 (8.1-31.9) | 1.1 (0.5-2) | 608.7 (269.5-1171.7) | 34.5 (15.8-65) |  | 61 (28-112.2) | 2.2 (1-4.1) | 2004.1 (903.9-3782.6) | 64.8 (30-120.7) |  | 3.04 (2.53-3.54) | 2.44 (2.14-2.75) |
| Northern Mariana Islands | 0.7 (0.3-1.2) | 5.7 (2.8-9.6) | 28.4 (12.1-54.3) | 151.6 (73.1-267.8) |  | 2.8 (1.3-4.8) | 7 (3.6-11.4) | 113.8 (50-203.8) | 214.7 (101.9-372.5) | | 0.88 (0.36-1.41) | 1.21 (0.76-1.66) |
| Norway | 61.9 (31.2-99.3) | 0.8 (0.4-1.3) | 2456.1 (1120-4333.4) | 37.4 (16.5-68.4) |  | 86 (44.1-136.7) | 0.8 (0.4-1.2) | 4306.8 (1974.6-7715.1) | 47 (21-86.3) |  | -0.39 (-0.76--0.02) | 0.31 (0.18-0.44) |
| Oman | 31.7 (15.9-53.4) | 6.6 (3.4-10.7) | 1067.1 (508.5-1839.5) | 168.6 (85.9-281) |  | 102.7 (60.2-155.1) | 10 (5.9-14.7) | 4481.8 (2338.8-7286.6) | 259.8 (147-395) |  | 1.87 (1.68-2.05) | 1.73 (1.55-1.91) |
| Pakistan | 1178.7 (566.6-1992) | 2.5 (1.2-4.2) | 37395.5 (17392.6-66894.4) | 69.1 (33.3-121.4) |  | 4128.4 (1957.1-7134.5) | 5.2 (2.6-8.8) | 135230.6 (61095.1-242172.1) | 133 (62.9-230.3) |  | 2.64 (2.33-2.94) | 2.32 (2.04-2.6) |
| Palau | 0.7 (0.3-1.2) | 8.2 (3.8-14.2) | 19.8 (8.6-36.9) | 205.9 (92.8-372.3) |  | 2.3 (1.1-4.2) | 13.7 (6.7-23.1) | 78.9 (34.4-147.9) | 366.1 (169.1-658.4) | | 1.68 (1.37-1.99) | 1.89 (1.59-2.19) |
| Palestine | 59.7 (31.5-93.7) | 8.1 (4.4-12.6) | 1572.9 (789.6-2532.3) | 190.2 (97-302.7) |  | 187.2 (104.8-291) | 10.9 (6.1-16.5) | 5958.5 (3128.4-9784.7) | 272 (150.9-434.5) |  | 0.97 (0.53-1.41) | 1.19 (0.88-1.5) |
| Panama | 6.7 (2.4-15.2) | 0.5 (0.2-1.1) | 227.4 (78.9-543.8) | 15.9 (5.6-37.2) |  | 42.8 (14.5-93.8) | 1 (0.3-2.2) | 1238.4 (408.1-2774.3) | 29.9 (9.8-67.3) |  | 2.04 (1.73-2.35) | 1.89 (1.73-2.05) |
| Papua New Guinea | 79.2 (32.5-157.4) | 5.7 (2.5-10.7) | 2613.3 (1023-5380.6) | 143.3 (59.9-283.6) |  | 295.1 (120.4-590.3) | 8.3 (3.8-15.5) | 10447.4 (4036.4-21123.5) | 216.2 (90-413.6) |  | 1.15 (0.96-1.34) | 1.31 (1.14-1.49) |
| Paraguay | 25.7 (9.5-50.6) | 1.3 (0.5-2.6) | 665.2 (229.2-1402.4) | 32.1 (11.4-66.3) |  | 173 (68.5-330.2) | 3.4 (1.4-6.5) | 4330.2 (1568.9-8644.8) | 81.4 (30.3-161.1) |  | 3.48 (3.02-3.95) | 3.33 (3.03-3.64) |
| Peru | 74.3 (27.4-143.4) | 0.7 (0.3-1.4) | 2432 (861.7-4964) | 21.4 (7.7-42.3) |  | 339.3 (142-624.7) | 1.1 (0.4-2) | 11907.6 (4736.2-23055.6) | 37.4 (14.9-72.3) |  | 1.79 (1.52-2.07) | 2.17 (1.99-2.35) |
| Philippines | 206.5 (77.1-432.6) | 1.2 (0.4-2.4) | 5433.8 (2018.3-12189.7) | 22.7 (8.6-47.3) |  | 615.6 (222.8-1369.2) | 1 (0.4-2.2) | 17559.9 (6262.2-39452.8) | 25.1 (9.2-55.3) |  | -0.57 (-0.81--0.34) | 0.35 (0.19-0.51) |
| Poland | 397.9 (193.8-696.9) | 0.9 (0.4-1.6) | 18899.6 (8560.8-34518.1) | 43.3 (19.7-79.5) |  | 681.6 (330.7-1156.1) | 0.9 (0.4-1.5) | 33465.8 (15482.4-61410.8) | 49.2 (22.1-90.5) |  | 0.21 (-0.05-0.48) | 0.62 (0.29-0.95) |
| Portugal | 360.2 (182.2-568.5) | 2.7 (1.4-4.1) | 10900.5 (5140.3-18489.4) | 78.3 (37.2-133.8) |  | 667.4 (364.2-1009.5) | 2.2 (1.2-3.4) | 21236.9 (10851.5-35392) | 90.7 (44.3-156.5) |  | -1.33 (-1.73--0.93) | -0.04 (-0.37-0.3) |
| Puerto Rico | 222.8 (125.7-336.3) | 6.4 (3.6-9.6) | 6810.3 (3647.9-11213.6) | 189.2 (101.2-310.8) | | 474.5 (260.4-745.3) | 5.9 (3.2-9.4) | 14828.3 (8021.3-24407.5) | 223.1 (115.5-374.1) | | -0.62 (-0.95--0.29) | 0.46 (0.16-0.77) |
| Qatar | 10.1 (5.6-15.7) | 19.3 (11.1-29) | 398.7 (205.3-660.3) | 413.6 (229.5-624.9) | | 56.6 (30.7-88.3) | 22.5 (12.8-33.4) | 4838.2 (2446.1-8160.4) | 517 (286.2-782) |  | 0.95 (0.45-1.45) | 1.03 (0.56-1.5) |
| Republic of Korea | 391.3 (157.9-752.6) | 1.6 (0.7-2.9) | 15511.4 (5475.5-30950) | 52.5 (20.1-100.7) |  | 1439.1 (668.3-2381) | 1.7 (0.8-2.8) | 46942.8 (19594-87140.3) | 53.3 (22.5-98.9) |  | -0.56 (-1.48-0.36) | -0.65 (-1.21--0.08) |
| Republic of Moldova | 11.4 (5.1-21.2) | 0.3 (0.1-0.5) | 651.7 (273.1-1263.8) | 15.7 (6.8-30.3) |  | 14.5 (7-26) | 0.2 (0.1-0.4) | 1111.5 (486.8-2165.3) | 19.1 (8.3-37.6) |  | -1.2 (-1.49--0.92) | 0.54 (0.43-0.65) |
| Romania | 102.5 (48.3-185.5) | 0.4 (0.2-0.7) | 5430.4 (2375.5-10328.8) | 19.7 (8.8-37.8) |  | 183.2 (87.6-318.1) | 0.5 (0.2-0.8) | 11080.8 (5171.2-20436.1) | 30.3 (13.6-57.1) |  | 0.7 (0.28-1.11) | 1.57 (1.33-1.81) |
| Russian Federation | 361.8 (171.8-654.6) | 0.2 (0.1-0.4) | 19045.5 (8506.7-35473.5) | 11 (4.9-20.2) |  | 1188.7 (550.4-2113.4) | 0.5 (0.2-0.9) | 42801.4 (20106.4-77644.4) | 17.7 (8.3-32.4) |  | 3.22 (2.41-4.04) | 2.18 (1.87-2.5) |
| Rwanda | 23.6 (9-55.6) | 1.1 (0.4-2.4) | 616.5 (232.8-1500) | 23.5 (8.9-55.4) |  | 38.4 (14.1-83.5) | 0.9 (0.4-1.9) | 1024.1 (392.9-2366.6) | 19.9 (7.7-44.5) |  | -1.05 (-1.27--0.83) | -1.14 (-1.38--0.9) |
| Saint Kitts and Nevis | 3.3 (1.7-5.3) | 9.1 (4.7-14.6) | 85.2 (40.9-141.7) | 231.1 (109.7-394.1) | | 4.1 (2.1-6.7) | 7.5 (4.1-12) | 150.3 (69.9-263.1) | 230.1 (113.5-391.9) | | -0.39 (-0.56--0.23) | -0.08 (-0.2-0.04) |
| Saint Lucia | 8.6 (4.5-13.9) | 10.8 (5.7-17.4) | 236.8 (112.3-400.6) | 275.5 (130.2-464.7) | | 17.7 (9.5-28.4) | 8.7 (4.7-13.8) | 588.8 (290.8-1002.1) | 275.5 (137.8-465.1) | | -1.73 (-2.09--1.37) | -0.61 (-0.81--0.4) |
| Saint Vincent and the Grenadines | 7.4 (3.9-11.9) | 11 (5.7-17.5) | 183 (88.5-310.2) | 258.5 (125.7-438.8) | | 14.2 (7.6-22.4) | 11.3 (6.1-17.6) | 418.2 (206.9-711.5) | 311.8 (156.3-526) |  | -0.26 (-0.47--0.04) | 0.28 (0.11-0.45) |
| Samoa | 5.8 (2.8-10.4) | 7.7 (3.8-13.4) | 172.7 (81-318.5) | 200.1 (94.5-362.3) |  | 13.6 (6.7-23.2) | 10.5 (5.3-17.5) | 434.9 (199.4-773.1) | 295 (140.3-513.2) |  | 0.86 (0.51-1.21) | 1.11 (0.76-1.47) |
| San Marino | 0.3 (0.2-0.5) | 1 (0.5-1.6) | 10.8 (5.1-18.9) | 33 (15.6-58.3) |  | 0.7 (0.3-1.1) | 0.8 (0.4-1.4) | 30.6 (14.6-54.3) | 49.5 (22.5-90.3) |  | -0.33 (-0.44--0.22) | 1.5 (1.45-1.55) |
| Sao Tome and Principe | 0.4 (0.1-0.7) | 0.7 (0.3-1.3) | 15.1 (6.2-29.8) | 24.9 (10.2-48.3) |  | 0.8 (0.3-1.5) | 1 (0.4-1.9) | 38.5 (15.5-77.2) | 39.7 (16.7-78) |  | 1.28 (1.2-1.36) | 1.5 (1.4-1.6) |
| Saudi Arabia | 220.7 (119.8-349.3) | 4.5 (2.5-6.9) | 10060.4 (5253.1-16061.6) | 156.7 (85.1-244.1) |  | 569 (324.4-847) | 3.8 (2.2-5.5) | 52641.3 (28764.7-80782.3) | 213.8 (120.6-324.1) | | -0.88 (-1.11--0.65) | 1.51 (1.28-1.74) |
| Senegal | 63 (27.8-115.7) | 2.4 (1.1-4.4) | 2100.4 (889-3955.5) | 68.8 (30.2-126.6) |  | 201.5 (90.2-358.3) | 3.3 (1.5-5.8) | 6855.4 (2882.9-12983.3) | 96.1 (42.1-176.4) |  | 1.01 (0.76-1.25) | 1.19 (0.96-1.42) |
| Serbia | 101.5 (47.5-188.2) | 1 (0.5-1.9) | 3696.4 (1629.1-7078.1) | 34 (15.5-64.3) |  | 232.7 (107-422.4) | 1.5 (0.7-2.6) | 7714.6 (3453.9-14572.5) | 48.2 (21.4-91.6) |  | 1.56 (1.32-1.81) | 1.3 (1.18-1.41) |
| Seychelles | 0.6 (0.3-1) | 1 (0.5-1.9) | 21.3 (9-40.9) | 37.7 (15.8-72.3) |  | 1.8 (0.8-3.3) | 2 (0.9-3.6) | 85.8 (34.8-168.4) | 83.3 (35-159.7) |  | 2.21 (2.01-2.41) | 2.71 (2.56-2.87) |
| Sierra Leone | 24.2 (10.1-46.9) | 1.5 (0.6-2.9) | 573.7 (228.2-1178.4) | 32 (12.9-65.1) |  | 61.9 (25.8-114.5) | 2.2 (0.9-4.1) | 1655 (647.9-3231.2) | 50.6 (20.6-95.3) |  | 1.54 (1.3-1.78) | 1.78 (1.58-1.98) |
| Singapore | 33.1 (13.9-59.3) | 1.8 (0.8-3.1) | 1433.3 (529.9-2806.4) | 65.6 (25.4-126.7) |  | 19.4 (9-32.3) | 0.3 (0.1-0.5) | 3005 (1150.2-6280.1) | 38.1 (14.9-78.5) |  | -6.66 (-7.72--5.6) | -1.86 (-2.12--1.6) |
| Slovakia | 44.5 (21.5-82.8) | 0.8 (0.4-1.4) | 1630.1 (724.8-3121.7) | 27.4 (12.1-52.2) |  | 53.8 (25.1-97.2) | 0.6 (0.3-1.1) | 2824.8 (1254.5-5366.6) | 31.1 (13.7-59.9) |  | -0.72 (-1.03--0.41) | 0.37 (0.06-0.69) |
| Slovenia | 15.3 (7.1-27.6) | 0.7 (0.3-1.2) | 585.4 (258.6-1089.9) | 24.2 (10.8-45.1) |  | 27.7 (12.7-50) | 0.5 (0.2-1) | 1255.7 (551.7-2383.7) | 29.1 (12.4-56.9) |  | -2.9 (-3.78--2) | -0.33 (-0.72-0.07) |
| Solomon Islands | 6.9 (2.8-15.2) | 5.7 (2.5-11.5) | 243.1 (90.8-536.9) | 162.6 (66.2-341.4) |  | 30.1 (12.1-58.4) | 11.3 (5.1-20.3) | 1091.7 (422.2-2224.4) | 317.3 (133.1-609) |  | 2.25 (1.95-2.56) | 2.24 (1.94-2.54) |
| Somalia | 15.7 (5.7-37.2) | 0.9 (0.3-2.1) | 446 (163.8-1090.1) | 20.8 (7.7-47.9) |  | 40.1 (14.8-90.4) | 0.9 (0.4-2) | 1219.8 (446.5-2799) | 21.6 (8.3-48) |  | 0.3 (0.22-0.39) | 0.32 (0.25-0.38) |
| South Africa | 828.5 (399.1-1378.6) | 4.4 (2.2-7.3) | 25499 (11856.8-43813.7) | 120.9 (57.4-206.5) |  | 2753.1 (1301.6-4583.4) | 7.3 (3.5-11.9) | 76053.8 (34547.3-130997.7) | 175.6 (80.9-297.7) |  | 2.36 (1.69-3.03) | 1.85 (1.27-2.43) |
| South Sudan | 15.5 (5.9-32.8) | 0.9 (0.3-1.8) | 388.4 (144.8-850.5) | 18.3 (6.8-38.8) |  | 24.7 (9.6-52.5) | 0.9 (0.4-1.9) | 681.2 (256.5-1515.7) | 20.9 (7.9-44) |  | 0.36 (0.33-0.39) | 0.5 (0.47-0.53) |
| Spain | 1186.6 (587.6-1925.6) | 2.2 (1.1-3.6) | 36148 (16601.9-64257.7) | 65.9 (30.2-118) |  | 1530.3 (809.5-2425.4) | 1.2 (0.6-1.8) | 60215.2 (28032.9-107975.6) | 63.5 (28.2-119.1) |  | -2.51 (-2.71--2.3) | -0.56 (-0.8--0.32) |
| Sri Lanka | 106.7 (42.2-212.2) | 1.4 (0.6-2.8) | 3313.4 (1252.4-6722.6) | 36.8 (14-72.4) |  | 716.8 (279.6-1411.6) | 3.6 (1.4-7) | 19734.8 (7503-39080.5) | 83.4 (32.6-162.9) |  | 4.09 (3.61-4.57) | 3.62 (3.29-3.95) |
| Sudan | 177.7 (91.2-283.4) | 2.2 (1.1-3.5) | 9313.5 (5129.7-14615.5) | 96.6 (53.6-150.3) |  | 491.1 (256.8-811.2) | 3 (1.6-4.8) | 34426.6 (19077.3-53832.8) | 167.9 (93.7-258.1) |  | 1.41 (1.23-1.59) | 2.13 (2.04-2.22) |
| Suriname | 10.7 (5.5-17.4) | 4.5 (2.3-7.2) | 369.2 (162.4-654.3) | 143.4 (65.4-246.9) |  | 34.2 (17.7-55.8) | 6.1 (3.2-9.8) | 1426.7 (667.7-2496.5) | 236.1 (114.1-406.4) | | 1.09 (0.78-1.4) | 1.85 (1.67-2.03) |
| Sweden | 148.1 (70.8-250.5) | 0.9 (0.4-1.5) | 4125.7 (1804.9-7546.3) | 27.4 (11.5-51.4) |  | 221.2 (101.4-379.2) | 0.8 (0.4-1.4) | 6566.2 (2795.8-12165.4) | 31.8 (13-61.2) |  | -0.97 (-1.25--0.69) | -0.5 (-0.94--0.07) |
| Switzerland | 206.2 (106.7-322.8) | 1.8 (0.9-2.8) | 5117.6 (2543.3-8654.8) | 47.9 (23.1-82) |  | 203.3 (105.4-312.3) | 0.9 (0.5-1.4) | 7749.6 (3660.7-13724.1) | 45.3 (20.4-82.9) |  | -3.07 (-3.37--2.76) | -0.51 (-0.61--0.4) |
| Syrian Arab Republic | 128.6 (70.9-210) | 2.9 (1.6-4.6) | 5496.7 (2672.1-9325.6) | 103.8 (53.9-171.6) |  | 247.7 (131.4-407.3) | 2.6 (1.4-4.1) | 15572.4 (7664.3-26736.5) | 126.3 (65.3-209.8) |  | -1.07 (-1.4--0.74) | 0.23 (0.03-0.43) |
| Taiwan (Province of China) | 299.6 (132.1-548.2) | 2.4 (1.1-4.3) | 8050.4 (3324.1-15713.4) | 56.4 (24.6-105.6) |  | 1084.8 (508.6-1903.8) | 2.7 (1.3-4.7) | 26444.3 (11816.1-48560.1) | 66 (29.4-120.3) |  | -0.69 (-1.22--0.15) | -0.24 (-0.62-0.15) |
| Tajikistan | 10.4 (4.5-20.8) | 0.4 (0.2-0.8) | 417.8 (169.9-855) | 15 (6.2-30.1) |  | 48.5 (19.9-99.7) | 1.3 (0.6-2.5) | 1871.6 (723.5-3972) | 40.8 (17.2-80.1) |  | 4.83 (4.5-5.15) | 3.86 (3.61-4.11) |
| Thailand | 421.3 (169.4-811.6) | 1.5 (0.6-2.8) | 12692.5 (4991.3-25607) | 39.8 (16.5-75.6) |  | 1343 (572.6-2521.9) | 1.4 (0.6-2.5) | 49234.1 (20660.5-94322.7) | 48.6 (20.5-93.1) |  | -1.13 (-1.56--0.7) | 0.1 (-0.2-0.4) |
| Timor-Leste | 2.4 (0.9-5) | 1.3 (0.5-2.6) | 71.6 (26.4-155.9) | 32.4 (12.9-65.7) |  | 9.3 (3.5-19.2) | 1.5 (0.6-3) | 317.4 (120.3-653.7) | 43.6 (17.1-86.9) |  | 0.47 (0.24-0.7) | 0.91 (0.7-1.12) |
| Togo | 14.7 (6.1-28.8) | 1.6 (0.7-3.1) | 405 (155.7-811.4) | 36.7 (14.9-70.8) |  | 58 (23.3-113.2) | 2.3 (1-4.3) | 1730.4 (658.8-3583.4) | 53.3 (21.6-101.8) |  | 1.08 (0.83-1.32) | 1.18 (0.96-1.4) |
| Tokelau | 0.1 (0-0.2) | 7.9 (3.8-13.7) | 2.7 (1.2-4.9) | 205 (93-373.2) |  | 0.1 (0.1-0.2) | 11.3 (5.5-20) | 4 (1.8-7.3) | 301.5 (139.4-548.9) | | 1.18 (0.91-1.46) | 1.25 (1.01-1.49) |
| Tonga | 4.6 (2.2-7.9) | 9.5 (4.6-16.1) | 129.3 (58-233.7) | 234 (108.4-414.2) |  | 9.1 (4.6-15.8) | 11.9 (6-20.5) | 241.7 (113-429.4) | 306.2 (144-538) |  | 0.79 (0.53-1.06) | 0.82 (0.56-1.08) |
| Trinidad and Tobago | 128.5 (69.2-203.3) | 16.5 (9-25.9) | 3733.8 (1820-6274.8) | 447.2 (221.3-746) |  | 275.6 (146.4-453.9) | 15.2 (8.2-24.8) | 8337.3 (4346.7-13879.3) | 444.6 (232.8-733.9) | | -0.65 (-0.86--0.44) | -0.39 (-0.56--0.22) |
| Tunisia | 29 (12.4-55.2) | 0.8 (0.3-1.4) | 1427.7 (576.2-2791) | 31 (12.9-59.1) |  | 141.3 (61.9-260) | 1.3 (0.6-2.3) | 7662.7 (3229.2-14618.7) | 62.3 (26.5-117.2) |  | 1.76 (1.61-1.9) | 2.9 (2.73-3.07) |
| Turkey | 1609.4 (826.9-2587.5) | 5.2 (2.7-8.2) | 45979.5 (22423.2-77877.2) | 132.4 (66.5-218.6) |  | 2754.7 (1535.7-4254.3) | 3.4 (1.9-5.2) | 100813.6 (52943.1-167537.6) | 115.1 (61-189.5) |  | -1.27 (-1.55--0.98) | 0.01 (-0.33-0.35) |
| Turkmenistan | 6.6 (2.9-12.8) | 0.4 (0.2-0.7) | 291 (118.2-599.3) | 15.8 (6.7-30.9) |  | 29.6 (12.4-57.5) | 0.8 (0.4-1.6) | 1435.2 (567.2-2863) | 36.4 (15.4-70.7) |  | 1.85 (1.5-2.2) | 2.57 (2.37-2.76) |
| Tuvalu | 0.5 (0.2-0.9) | 8.6 (4-15.2) | 14.5 (5.9-28.1) | 214.9 (93-408) |  | 1.1 (0.5-2) | 12.3 (5.8-22.4) | 31.8 (13.6-60.9) | 316 (143.6-590) |  | 1.09 (0.8-1.38) | 1.2 (0.94-1.46) |
| Uganda | 42.8 (15.9-97.6) | 0.9 (0.3-2) | 1119.3 (414.8-2628.6) | 19.7 (7.4-45) |  | 103.7 (40-222.7) | 1 (0.4-2.2) | 2934.3 (1109.1-6757.3) | 24.1 (9.3-53.2) |  | 0.33 (0.18-0.47) | 0.55 (0.39-0.7) |
| Ukraine | 94.9 (44-179.9) | 0.1 (0.1-0.3) | 5844.1 (2471.5-11629.2) | 8.4 (3.5-16.7) |  | 93.1 (42-177.8) | 0.1 (0.1-0.2) | 7145.1 (2964.2-14215.9) | 9.3 (3.8-19) |  | -1.33 (-1.58--1.07) | 0.22 (0.14-0.29) |
| United Arab Emirates | 32 (17.6-48.8) | 13.8 (7.7-20.7) | 1474.6 (838.9-2259.3) | 342.2 (195.4-502.9) | | 244.5 (130.4-397.7) | 10.4 (5.8-15.9) | 18303.2 (10026-29091.6) | 353 (199-537.4) |  | -0.96 (-1.65--0.26) | 0.11 (-0.35-0.58) |
| United Kingdom | 967.8 (501.2-1522.9) | 1 (0.5-1.6) | 36159 (17199.5-61902.5) | 41.2 (19.2-72.2) |  | 909.7 (490.5-1406.6) | 0.6 (0.3-1) | 76390.6 (36467.6-137989.7) | 68.5 (31-128.9) |  | -2.01 (-2.17--1.84) | 1.98 (1.86-2.1) |
| United Republic of Tanzania | 64.7 (25.5-139.6) | 0.8 (0.3-1.7) | 1555.8 (607.1-3436.3) | 16.4 (6.5-35) |  | 177 (66.8-377.3) | 1 (0.4-2) | 4435.6 (1681-9814.7) | 20.6 (7.8-43.8) |  | 0.55 (0.51-0.59) | 0.93 (0.89-0.98) |
| United States of America | 4209.2 (1928.1-7259.2) | 1.3 (0.6-2.2) | 151735 (65061.3-282720.4) | 48.4 (20.3-91.3) |  | 5447.1 (2386.9-9995.9) | 0.9 (0.4-1.7) | 268536.2 (112240.1-521559.6) | 50.6 (20.8-99.8) |  | -1.7 (-2.33--1.08) | 0.52 (0.29-0.75) |
| United States Virgin Islands | 2.7 (1.2-4.8) | 4.1 (1.8-7.1) | 77.5 (32.1-143.3) | 98.7 (42.2-176.5) |  | 7.2 (3.6-12.3) | 4.3 (2.2-7.1) | 224.5 (109.5-401.4) | 123.9 (60.4-220.6) |  | 0.16 (-0.08-0.4) | 0.74 (0.55-0.94) |
| Uruguay | 37.8 (12.5-78.5) | 1 (0.3-2) | 830.3 (269.3-1803.5) | 21.2 (6.9-45.9) |  | 72.7 (27.9-133.7) | 1.2 (0.5-2.2) | 2101 (790.3-4073.5) | 39.8 (14.5-79.1) |  | 0.85 (0.75-0.95) | 2.34 (2.11-2.57) |
| Uzbekistan | 32.6 (14.7-63.9) | 0.3 (0.1-0.6) | 1362 (556.7-2799.4) | 12.3 (5.1-24.4) |  | 265.6 (109.5-521.4) | 1.6 (0.7-3) | 9948 (3915.5-20252.1) | 50.3 (21.4-96.4) |  | 5.85 (5.26-6.44) | 5.05 (4.71-5.39) |
| Vanuatu | 1.4 (0.6-3) | 2.8 (1.2-5.8) | 46.2 (17.6-102.9) | 74.2 (30-155.3) |  | 7.3 (3.1-14.3) | 5.4 (2.3-10.3) | 230.8 (91.4-462.4) | 140 (57.9-274.4) |  | 2.35 (2.22-2.49) | 2.2 (2.08-2.31) |
| Venezuela (Bolivarian Republic of) | 73.5 (25.4-167.7) | 0.9 (0.3-2) | 2363.8 (802.8-5479.4) | 25.6 (8.7-59.1) |  | 330.7 (108.8-725.6) | 1.3 (0.4-2.7) | 9863.7 (3303.8-22527.3) | 35.3 (11.8-79.7) |  | 0.97 (0.72-1.21) | 0.97 (0.76-1.19) |
| Viet Nam | 474.2 (178.4-962.6) | 1.4 (0.5-2.8) | 10555.8 (3892.4-21853) | 28.9 (10.7-59.9) |  | 1302.7 (515.2-2537.8) | 1.8 (0.7-3.5) | 32308.1 (12281.3-67175.6) | 40.2 (15.5-81.6) |  | 0.92 (0.75-1.09) | 1.36 (1.21-1.52) |
| Yemen | 62.2 (29.6-112.1) | 1.6 (0.8-2.9) | 2778.5 (1306.2-5093.8) | 58.4 (29-102.4) |  | 216.6 (105.9-390.7) | 2 (1-3.5) | 11456.4 (5159.5-20999.3) | 86.6 (41.9-151.2) |  | 1.05 (0.9-1.21) | 1.56 (1.46-1.66) |
| Zambia | 42.5 (16.6-86.1) | 2 (0.8-4) | 1190.7 (452.6-2526.7) | 45.9 (18.2-93.5) |  | 110.5 (43.4-220.5) | 2.3 (0.9-4.4) | 3323 (1259.4-6897.1) | 54.9 (21.8-110.5) |  | 0.25 (0.07-0.44) | 0.46 (0.31-0.6) |
| Zimbabwe | 34.4 (14.2-70.6) | 1.1 (0.5-2.3) | 1026.9 (388.8-2152.9) | 28 (11-57.7) |  | 102.6 (39.7-214.7) | 1.9 (0.8-3.9) | 3162.2 (1183.7-6911.4) | 49.5 (19.3-103.8) |  | 2.16 (1.81-2.5) | 2.21 (1.96-2.46) |

ASMR: age-standardized mortality rate; ASDR: age-standardized disability-adjusted life year rate; EAPC: estimated annual percentage change.
