# Supplementary figures and images for: Global trends in burden of type 2 diabetes attributable to physical inactivity across 204 countries and territories, 1990-2019
Source: Front Endocrinol (Lausanne). 2024 Feb 26;15:1343002. doi: 10.3389/fendo.2024.1343002 (PMC10925666; doi:10.3389/fendo.2024.1343002)

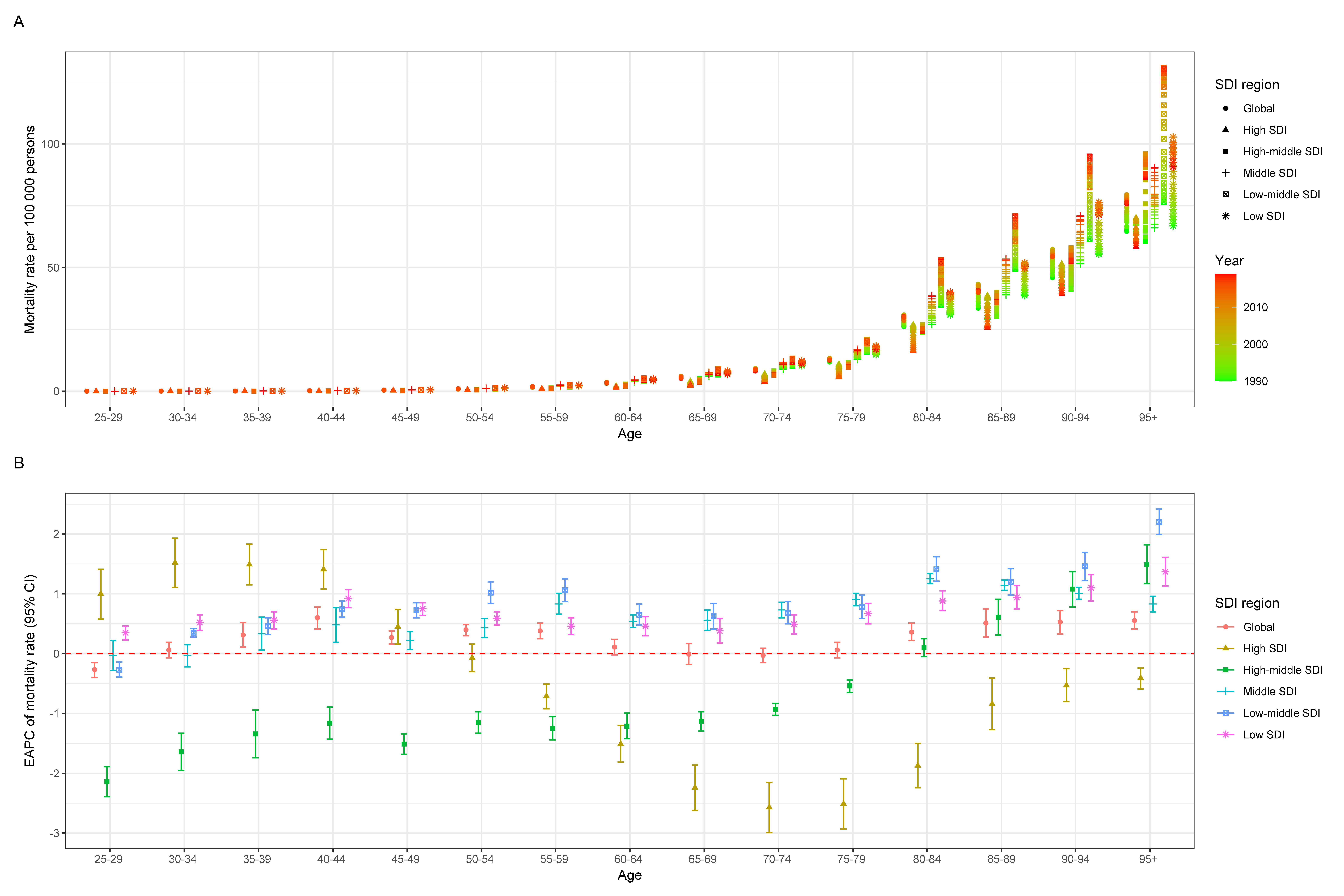

Supplement: Supplementary file 2 [file Image_1.tif]

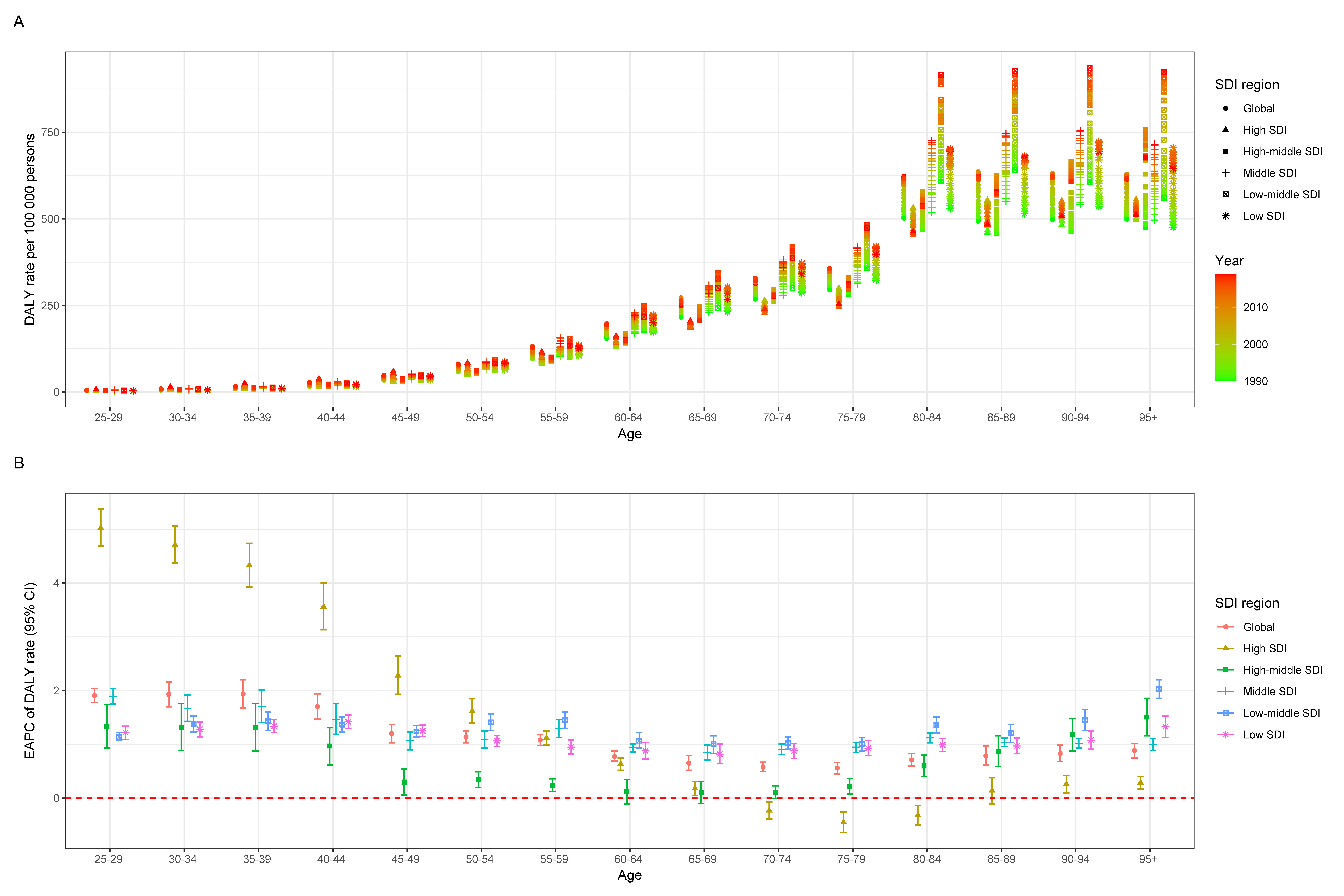

Supplement: Supplementary file 3 [file Image_2.tif]
